# Mitochondrial Respiratory Supercomplex Assembly Factor COX7RP Contributes to Lifespan Extension in Mice

**DOI:** 10.1111/acel.70294

**Published:** 2025-11-18

**Authors:** Kazuhiro Ikeda, Sachiko Shiba, Masataka Yokoyama, Masanori Fujimoto, Kuniko Horie, Tomoaki Tanaka, Satoshi Inoue

**Affiliations:** ^1^ Division of Systems Medicine & Gene Therapy, Faculty of Medicine Saitama Medical University Saitama Japan; ^2^ Laboratory of Physiology, Faculty of Pharmaceutical Sciences Josai University Saitama Japan; ^3^ Department of Molecular Diagnosis, Graduate School of Medicine Chiba University Chiba Japan; ^4^ Department of Systems Aging Science and Medicine Tokyo Metropolitan Institute for Geriatrics and Gerontology Tokyo Japan

**Keywords:** lifespan, metabolism, mitochondria, supercomplex, white adipose tissue

## Abstract

COX7RP is a critical factor that assembles mitochondrial respiratory chain complexes into supercomplexes, which is considered to modulate energy production efficiency. Whether COX7RP contributes to metabolic homeostasis and lifespan remains elusive. We here observed that *COX7RP*‐transgenic (*COX7RP*‐Tg) mice exhibit a phenotype characterized by a significant extension of lifespan. In addition, metabolic alterations were observed in *COX7RP*‐Tg mice, including lower blood glucose levels at 120 min during the glucose tolerance test (GTT) without a significant difference in the area under the curve (AUC), as well as reduced serum triglyceride (TG) and total cholesterol (TC) levels. Moreover, *COX7RP*‐Tg mice exhibited elevated ATP and nicotinamide adenine dinucleotide levels, reduced ROS production, and decreased senescence‐associated β‐galactosidase levels. Single‐nucleus RNA‐sequencing (snRNA‐seq) revealed that senescence‐associated secretory phenotype genes were downregulated in old *COX7RP*‐Tg white adipose tissue (WAT) compared with old WT WAT, particularly in adipocytes. This study provides a clue to the role of mitochondrial respiratory supercomplex assembly factor COX7RP in resistance to aging and longevity extension.

## Graphical abstract

Mitochondrial respiratory supercomplex assembly factor COX7RP improves metabolic homeostasis and contributes to lifespan extension in mice. The overexpression of COX7RP reduces white adipose tissue mass and downregulates senescence‐associated secreted phenotype‐related genes in the tissue, suggesting that COX7RP plays a role in preventing aging through mitochondrial functional remodeling.

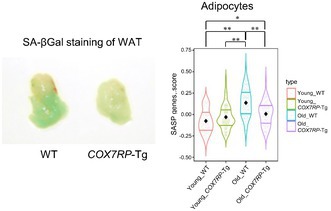

## 1 Introduction

Aging is a biological process promoting senescence of tissues and organs, subsequently resulting in an increase in the risk for morbidity and mortality (Kennedy et al. 2014). The impairment of metabolic and detoxicating pathways substantially contributes to the aging process, in which the cell organelles mitochondria play a crucial role (Balaban et al. 2005; Kauppila et al. 2017). The age‐related reduction of energy production in muscles is involved in the disorder of frailty, or sarcopenia (Goates et al. 2019; Longobucco et al. 2019). The imbalance of metabolic pathways can lead to the pathogenesis of metabolic syndrome with a cluster of medical conditions (Cornier et al. 2008). While mitochondria drive oxidative phosphorylation (OXPHOS) by utilizing a proton gradient generated by mitochondrial respiratory chain complexes (CI‐CIV) embedded in their inner membrane and generate energy in the form of ATP molecules predominantly via ATP synthase (CV), the organelles also elicit oxidative stress and produce reactive oxygen species (ROS) (Bennett et al. 2022). The excessive ROS production together with the decrease of ATP synthesis in the mitochondria can result in the disruption of calcium ion homeostasis, eventually inducing cell damage and causing various disorders such as aging, diabetes, and skeletal muscle diseases (Perez‐Campo et al. 1998). Moreover, the reduced activity of cytochrome *c* oxidase (COX) in cardiomyocytes and senescent cells is associated with the age‐related alterations of mitochondrial phenotypes (Gómez and Hagen 2012; Müller‐Höcker 1989).

Accumulating evidence from experimental animal models and human clinical studies suggests that mitochondrial function is closely associated with both lifespan extension and age‐related decline. It is well established that aging is generally accompanied by a decline in mitochondrial function, which is attributed to mitochondrial DNA damage, increased oxidative stress, and deterioration of mitochondrial quality control mechanisms. These changes are characterized by reduced respiratory activity, altered mitochondrial dynamics, and increased production of ROS. The age‐related decline in mitochondrial function has been implicated in the pathogenesis of various aging‐associated diseases, including neurodegeneration, sarcopenia, and metabolic disorders (Sun et al. 2016). Nevertheless, the causal relationship between mitochondrial respiratory activity and lifespan remains largely unclear. In particular, while many studies have reported shortened lifespans in disease models and interventions to overcome them, evidence for lifespan or healthspan extension under non‐disease conditions remains limited—caloric restriction being one of the few well‐established interventions (Radovic et al. 2025). While age‐associated changes in the expression of several respiratory chain subunits have been reported (Chandra et al. 2022; Srivastava et al. 2020), it remains to be characterized which functional changes in metabolic pathways are directly related to lifespan, whether the enhancement or attenuation of oxidative phosphorylation (OXPHOS) promotes healthy aging, and to what extent tissue‐specific roles of mitochondrial function contribute to lifespan regulation (Radovic et al. 2025). Thus, elucidating mitochondrial function will advance our understanding of the molecular mechanisms involved in aging and age‐related diseases.

We previously demonstrated that cytochrome *c* oxidase subunit 7a related polypeptide (COX7RP), or COX7A2L, is a key molecule whose structure resembles that of mitochondrial respiratory enzyme COX subunit 7a (COX7A) in CIV (Ikeda et al. 2013) and promotes the assembly of supercomplexes (Ikeda et al. 2019). Lapuente‐Brun et al. (2013) independently identified COX7RP as a supercomplex assembly promoting factor I (SCAFI) (Cogliati et al. 2016) and Lobo‐Jarne et al. (2018) reported that COXRP regulates CIII biogenesis and promotes supercomplexes. The functional role of supercomplex formation is still controversial; however, in mammalian cells, the formation of mitochondrial respiratory supercomplexes, CIII_2_ + CIV or “respirasome” composed of CI + CIII_2_ + CIV, is considered to facilitate efficient energy generation (Vercellino and Sazanov 2021; Lopez‐Fabuel et al. 2016). Mammalian COX7RP is structurally required for the assembly of CIII_2_ + CIV, resulting in higher oxidoreductase activity than individual enzymes (Vercellino and Sazanov 2021). We demonstrated that COX7RP regulates energy metabolism in muscle by modulating muscle activities and brown adipose tissue (BAT) homeostasis (Ikeda et al. 2013). In *Cox7rp* knockout (KO) mice, the mice exhibited lower levels of blood glucose in the oral glucose tolerance test (OGTT) and insulin tolerance test (ITT) (Shiba et al. 2017). Intriguingly, *Cox7rp*KO mice showed low levels of blood glucose in the pyruvate tolerance test (PTT), indicating impaired gluconeogenesis. The ATP content was reduced in the line of *Cox7rp*KO mice compared with that of wild‐type (WT) mice. As analyzed by two‐dimensional blue native polyacrylamide gel electrophoresis (BN‐PAGE), the percentage of CIII subunit Risp in the supercomplex fraction was lower in *Cox7rp*KO mice liver compared to WT mice. Taken together, COX7RP has crucial roles in the regulation of energy production and glucose homeostasis in vivo.

Considering that insulin resistance is often associated with aging and that gain‐of‐function of COX7RP is assumed to enhance exercise endurance in mice and humans (Benegiamo et al. 2022; Ikeda et al. 2013), we next questioned whether COX7RP contributes to the improvement of age‐related conditions in vivo and the extension of lifespan, because mitochondrial supercomplexes are considered to be involved in aging and age‐related diseases (Novack et al. 2020), as exemplified by the alterations of supercomplex contents in aged rat brains cortex (Frenzel et al. 2010) and in aged rat skeletal muscles (Lombardi et al. 2009).

Here, we show that COX7RP is an important regulatory factor for metabolic homeostasis along with promoting mitochondrial respiratory supercomplex assembly, which enhances ATP synthesis and suppresses ROS production, as revealed by the phenotype of *COX7RP*‐Tg mice. Particularly, *COX7RP*‐Tg mice exhibit favorable metabolic characteristics in WAT, with lower expression of senescence‐associated genes.

## 2 Results

### 2.1 COX7RP Contributes to Lifespan Extension in Mice

**FIGURE 1:**
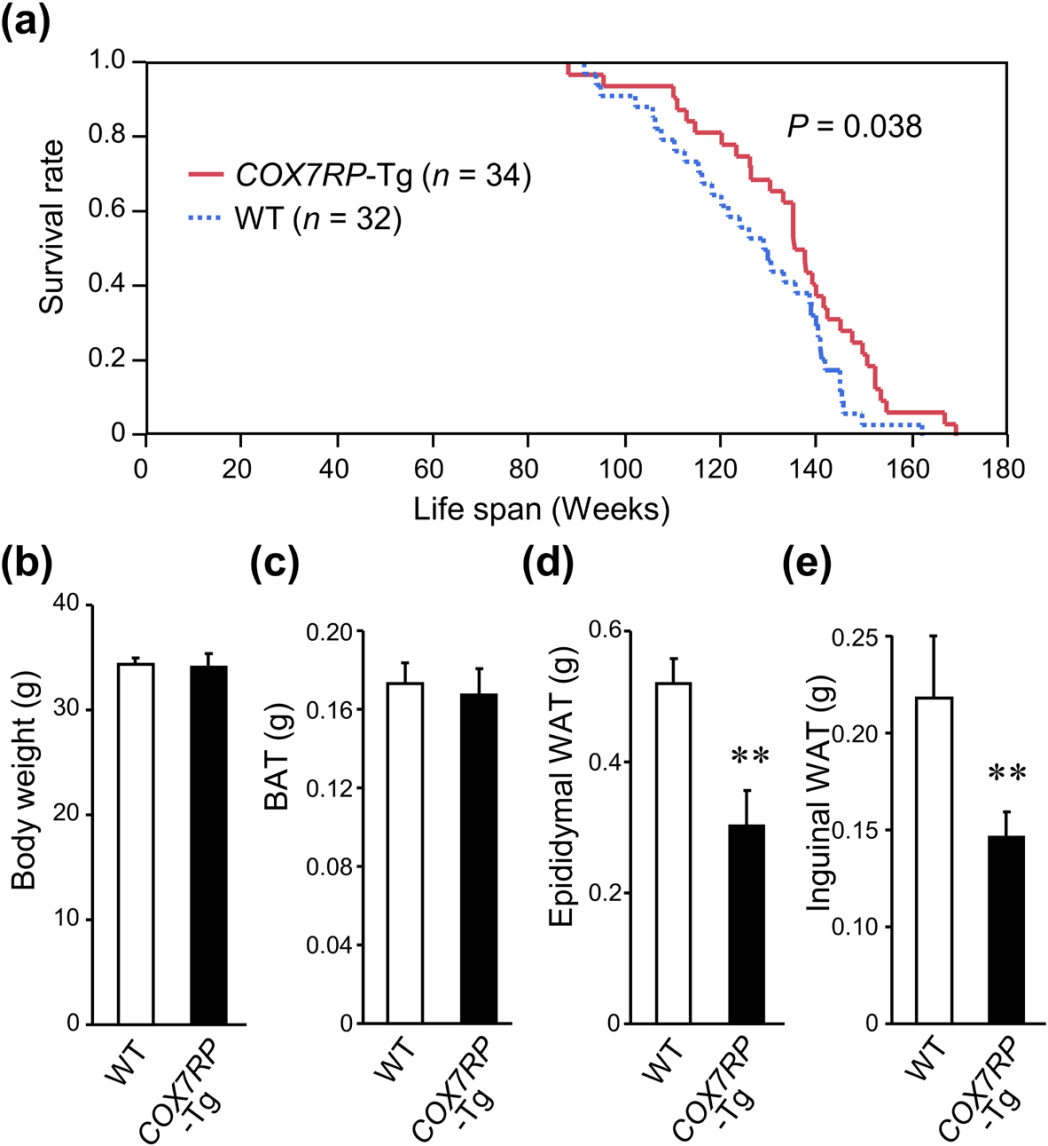
Male *COX7RP*‐Tg mice show increased lifespan and an effect on WAT. (a) Kaplan–Meier survival curves of *COX7RP*‐Tg (*n* = 34) and control wild‐type (WT) littermate (*n* = 32) mice. (b–e) Body weight (b) and tissue weight of brown adipose tissue (BAT) (c), epididymal white adipose tissue (WAT) (d), and inguinal WAT (e) of *COX7RP*‐Tg (*n* = 9) and WT (*n* = 9) mice at 10 months old. Data are presented as means ± SEM. Differences between *COX7RP*‐Tg and WT mice were analyzed using the log‐rank test (A) and a two‐tailed Student *t*‐test (b–e). *p < 0.05, **p < 0.01.

We previously showed that *COX7RP*‐Tg mice exhibited a phenotype with enhanced exercise endurance and increased COX activity in muscle (Ikeda et al. 2013). In this study, we observed that male *COX7RP*‐Tg mice showed a significantly prolonged lifespan compared with their male WT littermates based on Kaplan–Meier survival analysis (Figure 1a). The mean lifespans for the male WT and COX7RP‐Tg mice were 126.2 and 134.5 weeks, respectively, and thus the percent increase was 6.6%. Similarly, the median lifespans for the male WT and COX7RP‐Tg mice were 129.2 and 136.4 weeks, respectively, and thus the percent increase was 5.6%. We observed a slight increase in lifespan in female *COX7RP*‐Tg mice compared with the WT littermates; however, the difference was not statistically significant (*p* = 0.0946) (Figure S1). Thus, we focused on the analyses of male mice in the current study. While body weight and tissue weights of BAT, liver, pancreas, skeletal muscle, heart, and brain of male *COX7RP*‐Tg mice were not substantially different from those of WT mice at 10 months old, weights of epididymal and inguinal WAT pads were significantly lower than those of WT mice (Figure 1b–e and Figure S2). Consistent with the previous observation (Ikeda et al. 2013), the expression levels of *COX7RP* mRNA, combining endogenous mouse *Cox7rp* and exogenous human *COX7RP*, were increased in the epididymal WAT, quadriceps femoris muscle, and liver of *COX7RP*‐Tg mice (Figure S3). COX7RP protein levels in *COX7RP*‐Tg WAT and quadriceps tissues were upregulated by ~2 and ~4 folds, respectively, compared with those in WT tissues as analyzed by Western blotting (Figure S4).

### 2.2 Altered Lipid and Glucose Metabolism in *COX7RP* ‐Tg Mice

**FIGURE 2:**
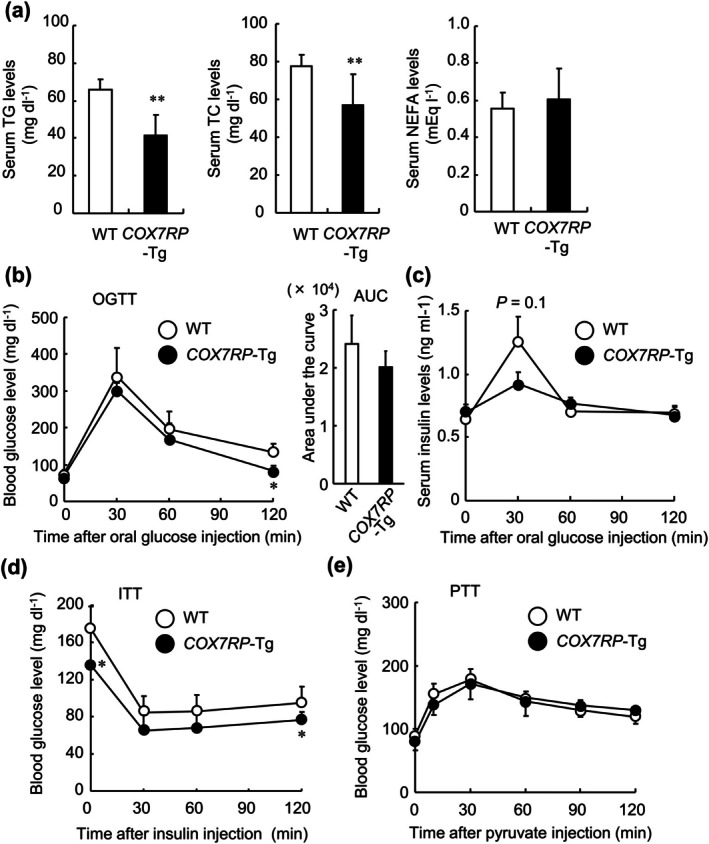
Improved fatty acid and glucose metabolism in male *COX7RP*‐Tg mice. (a) Serum levels of TG, TC, and NEFA in 10‐month‐old *COX7RP*‐Tg and WT mice. (b) Blood glucose levels during oral glucose tolerance test (OGTT) in 8‐month‐old *COX7RP*‐Tg and WT mice. The AUC for OGTT is shown in the right panel. (c) Plasma insulin levels during OGTT in 8‐month‐old mice. (d) Blood glucose levels during insulin tolerance test (ITT) in 8‐month‐old mice. (e) Blood glucose levels during pyruvate tolerance test (PTT) in 8‐month‐old mice. Data are presented as means ± SEM (*n* = 6). Differences between *COX7RP*‐Tg and WT mice were analyzed using a two‐tailed Student *t*‐test. *p < 0.05; **p < 0.01.

To understand the differences in WAT weights between *COX7RP*‐Tg and WT mice, we measured levels of serum lipids. Although no substantial difference was observed in non‐esterified fatty acid (NEFA), triglyceride (TG) and total cholesterol (TC) levels in 10‐month‐old *COX7RP*‐Tg mice appeared lower than those in age‐matched WT mice (Figure 2a) (Wilcox and Heimberg 1987). We next performed OGTT, ITT, and PTT in male *COX7RP*‐Tg and WT mice at 8 months of age (Figure 2b–e), a time point chosen to approximate middle age, as a difference in WAT weight was observed at 10 months. While the area under the curve (AUC) values for blood glucose in *COX7RP*‐Tg mice was similar to that of the age‐matched WT mice, a significant reduction in blood glucose levels at 120 min after oral glucose administration was observed in *COX7RP*‐Tg mice compared with that of WT mice, indicating a modest effect on glucose metabolism (Figure 2b). Plasma insulin levels also tended to be lower in *COX7RP*‐Tg mice compared with WT mice (Figure 2c). In ITT, *COX7RP*‐Tg mice displayed lower blood glucose levels at 0 and 120 min than WT mice (Figure 2d). PTT revealed that blood glucose levels were nearly equal in both genotype groups after pyruvate injection (Figure 2e). Overall, *COX7RP*‐Tg mice exhibit a phenotype with mildly altered lipid and glucose metabolism compared to the age‐matched WT mice.

### 2.3 Increased Oxygen Metabolism in *COX7RP* ‐Tg WAT and Skeletal Muscle

**FIGURE 3:**
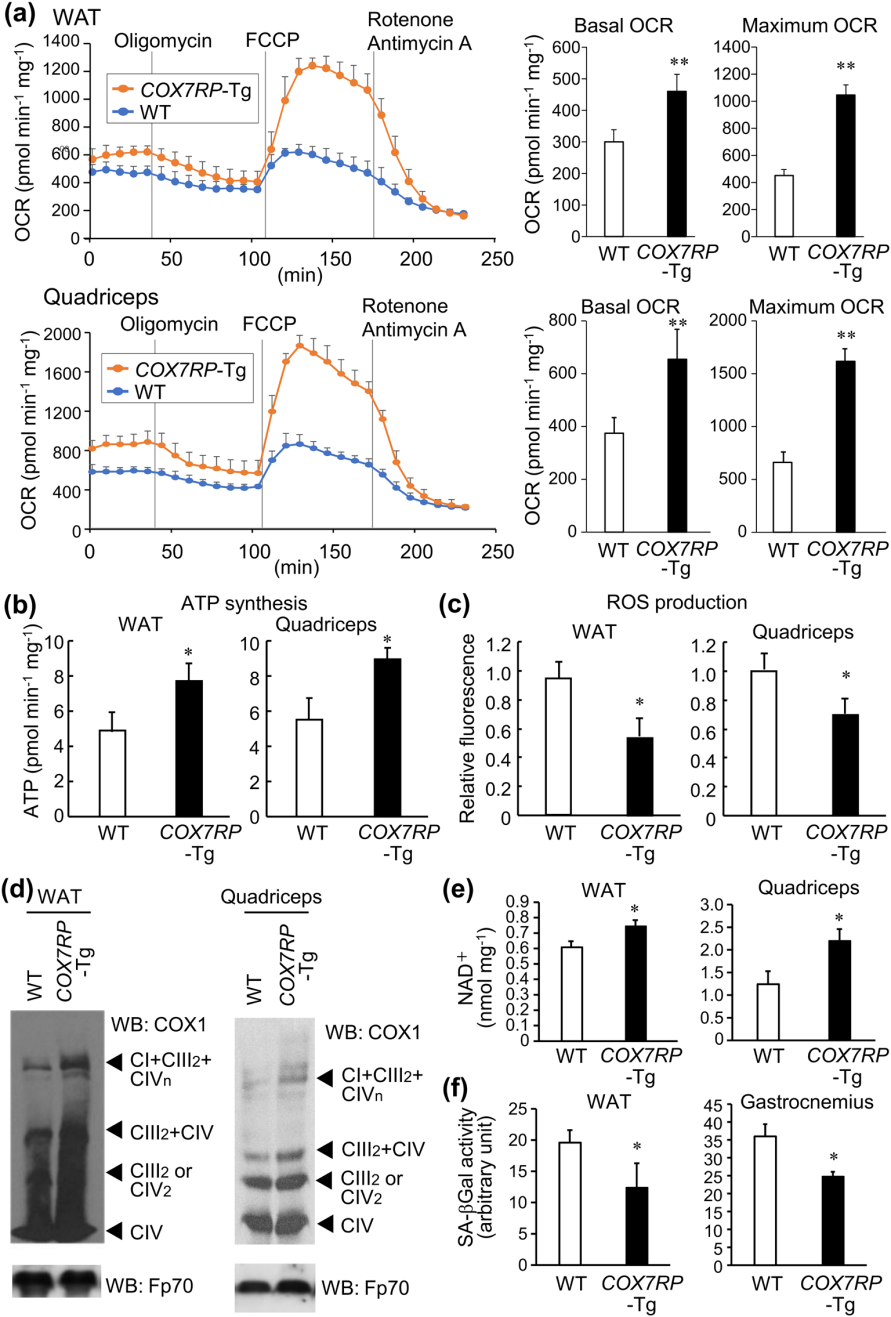
Increased mitochondrial respiratory supercomplex assembly and impaired senescence‐associated phenotypes in male *COX7RP*‐Tg mice. (a) Increased oxygen consumption rates (OCRs) in *COX7RP*‐Tg mice. OCR was measured in WAT and quadriceps femoris muscles dissected from 2‐year‐old *COX7RP*‐Tg and WT mice using a Flux analyzer with the sequential addition of oligomycin, carbonyl cyanide 4‐(trifluoromethoxy)phenylhydrazone (FCCP), and rotenone/antimycin A (*n* = 6). Basal and maximum OCRs were calculated after subtracting non‐mitochondrial OCR and the data are shown as means ± SD in the right panel (*n* = 6). (b and c) Increased ATP synthesis (b) and decreased ROS levels (c) in mitochondria from WAT and quadriceps femoris muscle in *COX7RP*‐Tg mice compared with WT mice at 2 years old. Data are presented as means ± SD (*n* = 3). (d) Mitochondrial respiratory supercomplex assembly is enhanced in WAT and quadriceps femoris muscle in *COX7RP*‐Tg mice. Mitochondrial proteins prepared from 2‐year‐old *COX7RP*‐Tg and WT mice were solubilized with digitonin (4 g g^−1^) and subjected to BN‐PAGE. Western blot analysis was performed with antibodies for COX1 and Fp70. Positions corresponding to CI+ CIII_2_ + CIV_n_, CIII_2_+ CIV, CIII_2_ or CIV_n_, and CIV are indicated. (e) Cellular nicotinamide adenine dinucleotide (NAD^+^) level is increased in WAT and quadriceps femoris muscle of *COX7RP*‐Tg mice at 2 years old. Data are presented as means ± SD (*n* = 3). (f) Senescence‐associated β‐galactosidase (SA‐β‐gal) activity is decreased in WAT and gastrocnemius muscle of *COX7RP*‐Tg mice at 2 years old. Data are presented as means ± SD (*n* = 3). Differences between *COX7RP*‐Tg and WT mice were analyzed using a two‐tailed Student *t*‐test. *p < 0.05; **p < 0.01.

We assessed respiratory states of WAT and quadriceps femoris muscle by analyzing oxygen consumption rates (OCRs) and extracellular acidification rates (ECARs), and showed that *COX7RP*‐Tg mice displayed elevated OCRs, particularly for basal and maximum respiration rates, in both tissues compared with WT mice (Figure 3a). Similarly, ECARs were increased in *COX7RP*‐Tg WAT and quadriceps femoris muscle (Figure S5). In addition, ATP synthesis and ROS levels were elevated and decreased in the mitochondria of quadriceps femoris muscle, respectively, from *COX7RP*‐Tg WAT compared with that of WT mice (Figure 3b,c).

### 2.4 Promotion of Mitochondrial Respiratory Supercomplex Assembly in *COX7RP* ‐Tg Mice

We next investigated the contribution of COX7RP to mitochondrial respiratory supercomplex assembly in WAT and muscle. Immunoblotting of BN‐PAGE gel for proteins obtained from digitonin‐solubilized mitochondria showed that the amounts of supercomplexes CIII_2_ + CIV and CI + CIII_2_ + CIV_n_ were increased in *COX7RP*‐Tg WAT and quadriceps femoris muscle while the expression levels of representative components involved in respiratory chain complexes, NDUFA9 for CI, RISP for CIII, and COX1 for CIV, were similar between the genotypes (Figure 3d and Figure S4). Intriguingly, aging‐related biomarkers nicotinamide adenine dinucleotide (NAD^+^) levels and senescence‐associated β‐galactosidase (SA‐β‐gal) activities were increased and decreased in *COX7RP*‐Tg WAT and muscle, respectively, compared with those of WT mice (Figure 3e,f, and Figure S6a).

### 2.5 SASP‐Associated Genes Are Repressed in Aged WAT

**FIGURE 4:**
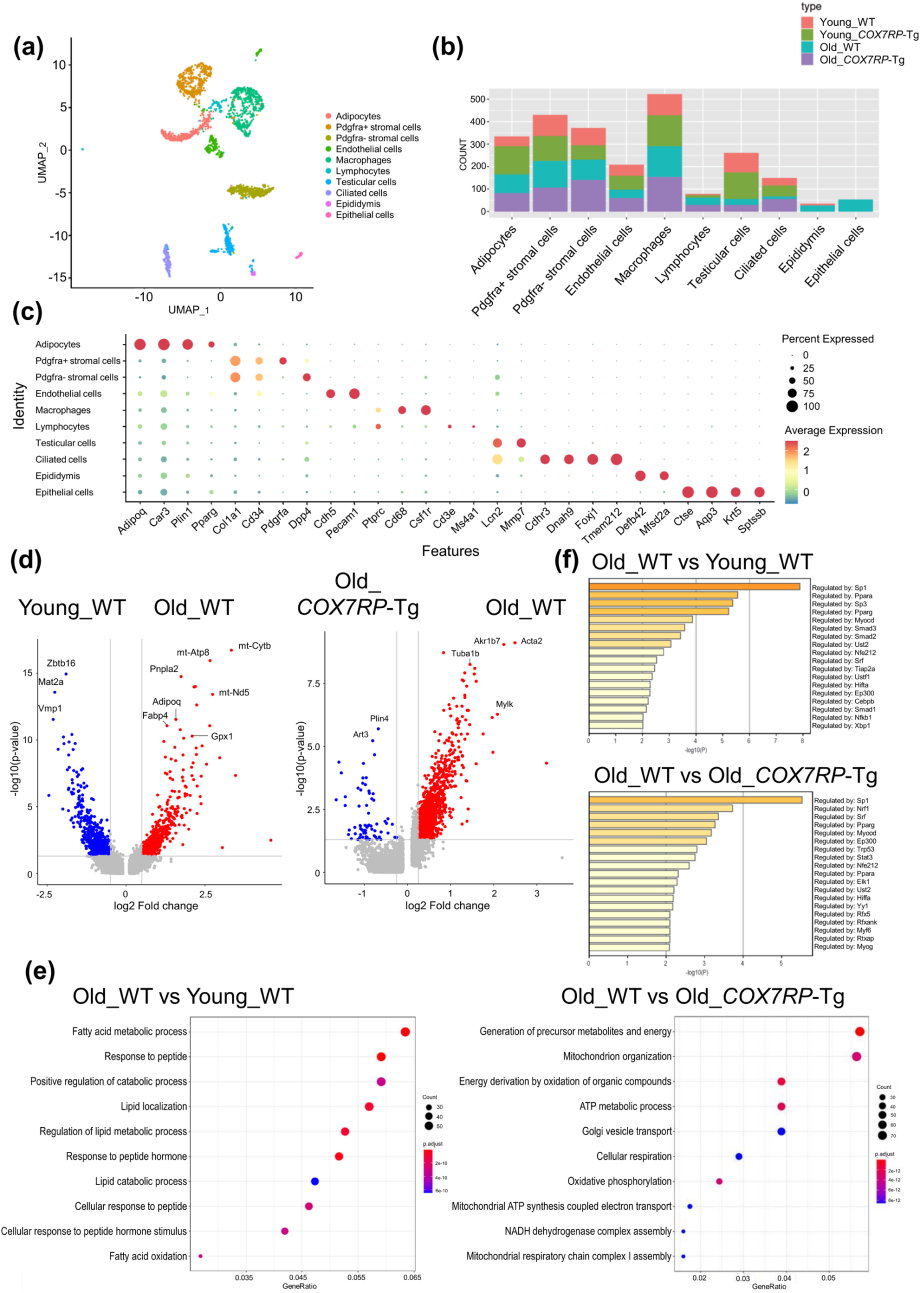
Landscape of WAT single cells obtained from young and old mice with either WT or *COX7RP*‐Tg phenotypes. (a) Uniform Manifold Approximation and Projection (UMAP) plots of snRNA‐seq data for WAT obtained from young (8‐week‐old) and old (2‐year‐old) mice with either WT or *COX7RP*‐Tg (Tg) phenotypes. (b) Proportions of WAT cell clusters from each distinct group. (c) Bubble heatmap showing expression levels of selected marker genes for each cell type. (d) Volcano plots for differentially expressed genes (DEGs) in comparisons between old WT versus young WT adipocytes (left panel) or between old WT versus old Tg adipocytes (right panel). DEGs were detected by using FindMarkers in Seurat (log_2_fc.threshold > 0.25 and *p* < 0.05). (e) Bubble plot of enriched pathways in the DEGs in comparisons between old WT versus young WT adipocytes (left panel) or between old WT versus old *COX7RP*‐Tg adipocytes (right panel). DEGs were defined as log_2_fc.threshold > 0.25. (f) Transcriptional Regulatory Relationships Unraveled by Sentence‐based Text‐mining (TRRUST) algorithm‐predicted enriched TF motifs in the regulatory regions of DEGs in comparisons between old WT versus young WT adipocytes (upper panel) or between old WT versus old Tg adipocytes (lower panel). DEGs detected in (e) were applied in Metascape.

**FIGURE 5:**
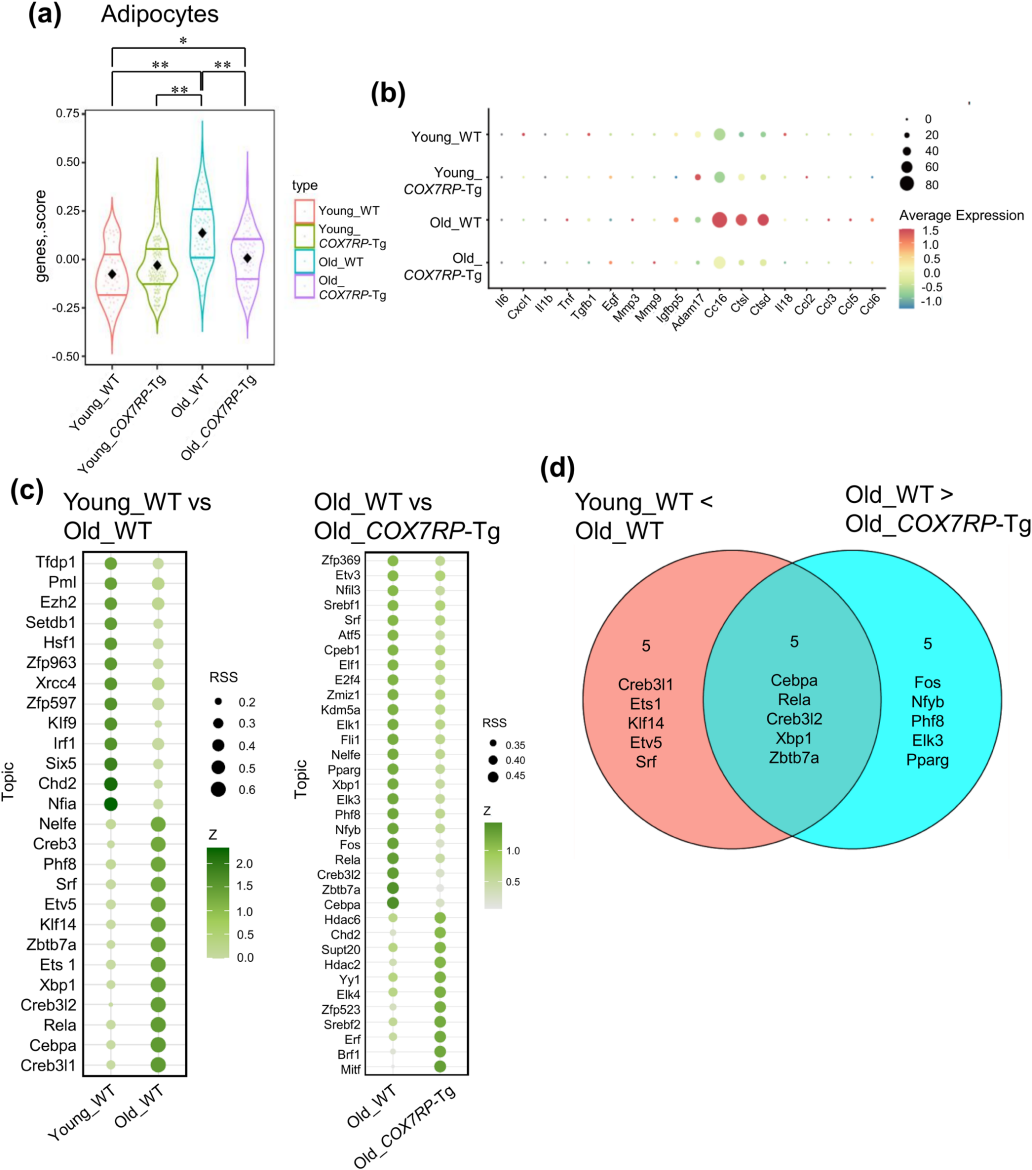
COX7RP represses SASP‐associated genes in aged adipocytes. (a and b) Violin (a) and dot (b) plots representing scores and expressions of selected SASP‐associated genes in adipocytes, respectively. The SASP‐associated genes score was calculated from the sum of *Z*‐scores for 18 SASP genes. A significance test was conducted using the one‐way ANOVA followed by the Tukey method, and *p* values were calculated for each group comparison. *p < 0.05; **p < 0.01. (c) Bubble heatmap for predominant TFs that potentially contribute to the regulation of SASP‐associated genes in each group of adipocytes predicted by single‐cell regulatory network inference and clustering (SCENIC) algorithm, in comparisons between old WT versus young WT adipocytes (left panel) or between old WT versus old *COX7RP*‐Tg adipocytes (right panel). According to the *Z*‐score and regulon specificity score (RSS), the top regulons were listed. (d) Top 10 predominant TFs relevant to the regulation of SASP‐associated genes for each comparison between old WT versus young WT adipocytes and between old WT versus old *COX7RP*‐Tg adipocytes, as shown in (c).

To further characterize the contribution of COX7RP to cell‐type transcriptome profiles in WAT, we performed snRNA‐seq analyses for epididymal WAT cell populations derived from four distinct groups: young (8‐week‐old) and old (2‐year‐old) mice with either WT or *COX7RP*‐Tg (Tg) genotypes. Cell populations from each group were classified into 10 clusters (Figure 4a–c and Figure S7). Among these cell clusters, adipocytes were defined as cell populations with high expression of adipocyte markers including *Adipoq*, *Car3*, *Plin1*, and *Pparg*. Stromal cells were classified into *Pdgfra*‐positive and ‐negative subclusters, both of which abundantly expressed *Col1a1* and *Cd34* while *Dpp4* was predominantly expressed in the *Pdgfra*‐negative subcluster. Blood vessel endothelial cells were defined as populations abundantly expressing *Pecam1* and *Cdh5*. Immune cells (*Ptprc* ^high^) were clustered into macrophages and lymphocytes. Macrophages highly expressed *Csf1r* and *Cd68*. On the other hand, the lymphocyte population included *Cd3e*‐positive or *Ms4a1*‐positive cells. Reproductive tissues were classified into the following categories: testicular cells as *Lcn2* ^high^/*Mmp7* ^high^, ciliated cells as *Cdhr3* ^high^/*Dnah9* ^high^/*Foxj1* ^high^/*Tmem212* ^high^, epididymis cells as *Defb42* ^high^/*Mfsb42* ^high^, and epithelial cells as *Ctse* ^high^/*Aqp3* ^high^/*Krt5* ^high^/*Sptssb* ^high^. The actual count for each cell cluster from the distinct groups is shown in Figure 4b. To dissect differentially expressed genes (DEGs) between young and old WT mice or between old WT and old Tg mice in snRNA‐seq, we depicted volcano plots for these comparisons (Figure 4d). Gene Ontology (GO) pathway analysis for upregulated DEGs revealed that fatty acid metabolism‐ and mitochondria‐related pathways were altered in old WT mice versus young WT mice and in old WT versus old Tg mice, respectively (Figure 4e). To predict upstream regulatory transcription factors (TFs) of the DEGs in adipocytes, we used Transcriptional Regulatory Relationships Unraveled by Sentence‐based Text‐mining (TRRUST) (Han et al. 2018) via Metascape (Zhou et al. 2019) (Figure 4f). TFs such as Sp1, Srf, Pparg, Myocd, and Ep300 were identified as correct upstream regulators with significant motif enrichment, likely predicting their contribution to the transcriptional regulation of DEGs in old WT mice versus young WT mice and in old WT versus old Tg mice. In the adipocyte cluster, higher expression of SASP‐associated genes was observed in old WT versus young WT mice whereas not in old *COX7RP*‐Tg versus young *COX7RP*‐Tg mice (Figure 5a,b and Figure S8). We selected prototypic SASP‐associated genes based on the gene expression data of WAT snRNA‐seq studies (Emont et al. 2022) and a list of SASP‐related genes originally proposed by Dr. Campisi Lab (Freund et al. 2010), including *Il6*, *Cxcl1*, *Il1b*, *Tnf*, *Tgfb1*, *Egf*, *Mmp3*, *Mmp9*, *Igfbp5*, *Adam17*, *Ctsb*, *Ctsl*, *Ctsd*, *Il18*, *Ccl2*, *Ccl3*, *Ccl5*, and *Ccl6*. Reverse transcriptase‐quantitative polymerase chain reaction (qRT‐PCR) verified that *Ccl5* and *Tnf* levels were significantly decreased in *COX7RP*‐Tg WAT compared with those of WT mice (Figure S6b). We also examined *p53* levels in *COX7RP*‐Tg and WT WATs, because p53 plays an important role in the development of insulin resistance and senescence‐like changes such as the expression of proinflammatory cytokines (Minamino et al. 2009). However, the expression levels of *p53* mRNA were not substantially different between these genotypes (Figure S6b), indicating that *p53* transcription is not primarily influenced by COX7RP overexpression. To further clarify the role of COX7RP in the adipocyte aging process, we applied the single‐cell regulatory network inference and clustering (SCENIC) algorithm (Aibar et al. 2017) to explore the potential upstream regulators for SASP‐associated genes with significant motif enrichment in adipocytes (Figure 5c and Figure S9). In particular, Cebpa, Rela, Creb3l2, Xbp1, and Zbtb7a were predicted as the top 5 upstream TFs putatively responsible for the SASP‐associated genes examined in the present study, based on the Venn diagram for the top 10 upstream TFs in comparisons between young WT versus old WT groups and between old WT versus old Tg groups (Figure 5d).

## 3 Discussion

The present study showed for the first time that an increase in COX7RP‐mediated mitochondrial respiratory supercomplex formation is associated with lifespan extension in mice, as well as enhanced cellular respiration rates ex vivo, and modest changes in glucose homeostasis and plasma lipid levels. COX7RP overexpression was associated with increases in mitochondrial respiration and ATP synthesis and a decrease in ROS production in WAT and skeletal muscles of *COX7RP*‐Tg mice compared with those of age‐matched WT mice. In terms of glucose metabolism, insulin sensitivity appeared to be modestly elevated in *COX7RP*‐Tg mice compared with age‐matched WT mice. Regarding lipid metabolism, reductions in serum TG and TC levels were observed in *COX7RP*‐Tg mice compared with age‐matched WT mice. Notably, snRNA‐seq analysis of WAT revealed a downregulation of SASP‐related genes in adipocytes from old *COX7RP*‐Tg mice compared to those from WT mice.

We observed that both *COX7RP*‐Tg (the present study) and *Cox7rp*KO (Shiba et al. 2017) mice exhibited lower blood glucose levels in OGTT and ITT compared with WT mice; however, we assume the underlying mechanisms for each phenotype may be distinct. In *COX7RP*‐Tg mice, the lower blood glucose levels in OGTT and ITT but not in PTT suggest enhanced insulin sensitivity without changes in gluconeogenesis. In contrast, middle‐aged *Cox7rp*KO mice showed lower blood glucose levels in PTT, indicating a progressive decline in gluconeogenic capacity with aging. Therefore, the lower blood glucose levels in ITT in *Cox7rp*KO mice would be attributable to the decreased gluconeogenesis rather than improvement of insulin sensitivity. Taken together, these observations suggest that the apparent improvement in GTT and ITT in *COX7RP*‐Tg and *Cox7rp*KO mice occurs through possible distinct mechanisms: enhanced insulin sensitivity in *COX7RP*‐Tg mice whereas altered gluconeogenesis in *Cox7rp*KO mice.

Our findings propose a novel concept that mitochondrial supercomplex formation prevents the SASP, a hallmark of senescent cells contributing to organismal aging (Zhang et al. 2022), based on the snRNA‐seq analysis of WAT from *COX7RP*‐Tg and WT mice. While WAT is a critical tissue with plasticity and its depot lipid can be transformed into metabolic energy, the understanding of single‐cell transcriptomics in WAT was limited due to the technical difficulty as WAT has tissue fragility and buoyancy (Maniyadath et al. 2023). We thus employed snRNA‐seq to characterize the multiple cell populations in WAT and successfully dissected the transcriptome of adipocytes based on the cell‐specific markers including *Adipoq*, *Car3*, *Plin1*, and *Pparg*, depicting the putative prominent gene signatures in each mouse group. Among SASP‐related genes, cathepsin genes were particularly enriched in old WT WAT versus young WT WAT, or versus old COX7RP‐Tg WAT. Cathepsins such as CTSD and CTSL are lysosomal proteinases that play physiological roles in various intracellular catabolism (Rovira et al. 2022; Zhao et al. 2024). Because SASP‐related proteins are initially derived from lysosomal exocytosis by senescent cells, cathepsins are included in SASP‐related genes. Of note, cathepsins may contribute to modulating microvascular transcytosis and the onset of diabetes (Zhao et al. 2024).

Structurally, COX7RP was characterized to promote the assembly of CIII₂CIV supercomplex by inserting its N‐terminus into CIII₂ and integrating its C‐terminus into CIV, thereby linking the two complexes (Vercellino and Sazanov 2021). The COX7RP‐dependent CIII₂CIV supercomplex assembly is potentially significant to facilitate respirasome (I + III_2_ + IV) formation, because the absence of COX7RP impairs the formation of these supercomplexes as shown by our group and others (Ikeda et al. 2013, 2019; Lapuente‐Brun et al. 2013), while some researchers reported that COX7RP does not affect respirasome formation (Pérez‐Pérez et al. 2016; Vercellino and Sazanov 2021). Nevertheless, COX7RP has physiological relevance in the segmentation of the electron transfer chain system into the simultaneous oxidation of multiple substrates at optimum rates as shown by a mouse experiment with or without COX7RP expression (Lapuente‐Brun et al. 2013). As a potential COX7RP‐mediated pathway that suppresses SASP gene expression, we speculate the involvement of SIRT1 and NF‐κB activities. NAD^+^‐dependent deacetylase SIRT1 has been shown to deacetylate NF‐κB, leading to the inhibition of NF‐κB transcription (Yeung et al. 2004). Because NAD^+^ levels decline during aging, decreased SIRT1 activity causes NF‐κB activation and upregulates SASP genes in an NF‐κB‐dependent manner (Yeung et al. 2004; Verdin 2015).

As NF‐κB has been reported as a critical TF for SASP‐related gene regulation (Lopes‐Paciencia et al. 2019), it is notable that the Rela subunit of the NF‐κB TF complex was shown as a potential upstream regulator for SASP‐related gene transcription based on our SCENIC analysis in adipocytes. The enhancement of NF‐κB signaling was observed in adipocytes of high‐fat diet‐fed mice, exhibiting adipose dysfunction and insulin resistance (Emont et al. 2022). Furthermore, NF‐κB signaling blockade in endothelial cells has anti‐aging relevance as it can target obesity‐ and age‐related insulin resistance (Hasegawa et al. 2012). Because ROS was shown to activate NF‐κB signaling and to cause DNA damage in senescent fibroblasts (Nelson et al. 2018), COX7RP‐mediated mitochondrial respiratory supercomplex assembly can repress NF‐κB‐dependent gene expression in *COX7RP*‐Tg adipocytes through the repression of ROS production. The reduction of ROS production may also result in the suppression of endoplasmic reticulum stress mediated by TFs such as Xbp1 and Creb3l2 (Murase et al. 2022), which we also identified as potential enriched pathways in old WT adipocytes. Zbtb7a has been recently shown as a putative aging modulator based on mouse and human data from multiple tissues (Bou Sleiman et al. 2020) and has oncogenic relevance involved in the glycolytic pathway.

We also predicted Sp1, Srf, Pparg, Myocd, and Ep300 as upstream regulatory TFs with significant motif enrichment of the DEGs in normal adipocyte aging based on the TRRUST platform. SP1 is known as a basal TF that represses adipocyte differentiation, whose transcriptional action is reversed by PPARγ and C/EBPα, master regulators of adipocyte differentiation (Roy et al. 2017; Tang et al. 1999). Serum response factor (SRF) is a driver TF for vascular smooth muscle development and cardiac myocyte differentiation, and Myocd is a coactivator for SRF transcriptional regulation (Wang et al. 2001). Notably, SRF signaling may also promote adipogenesis, because diacylglycerol O‐acyltransferase 2 (Dgat2), a critical enzyme for triglyceride synthesis, is a prototypic target of SRF (Swärd et al. 2016). In our snRNA‐seq, *Dgat2* was significantly upregulated in old WT adipocytes compared with old *COX7RP*‐Tg adipocytes (log_2_ fold change: 1.28, −log_10_(*p*‐value): 2.47E‐05 in old WT versus old *COX7RP*‐Tg). Interestingly, omega‐3 polyunsaturated fatty acid DHA, which is a PPARγ agonist with healthy benefits, downregulates SRF (Johnson et al. 2011). p300 is an essential coactivator that contributes to the inflammation‐induced transcription of cyclooxygenase‐2 (COX‐2), a key enzyme for prostaglandin synthesis (Deng et al. 2004). The upregulation of *Ptgs2*, a gene for COX‐2, was indeed observed in old WT adipocytes compared with old *COX7RP*‐Tg adipocytes (log_2_ fold change: 0.57, −log_10_(*p*‐value): 4.39E‐04 in old WT versus old *COX7RP*‐Tg). Taken together, COX7RP overexpression likely alters the adipocyte differentiation regulatory gene network by modulating the activity of critical TFs described above and maintains adipocyte homeostasis during aging.

Intriguingly, our snRNA‐seq indicated that OXPHOS‐related genes including components of CI, CIII, and CIV were relatively upregulated in old WT adipocytes compared with old *COX7RP*‐Tg adipocytes. During aging, the composition and fluidity of the inner mitochondrial membrane are known to significantly change: the age‐dependent alteration may cause difficulties in assembling respiratory complexes into supercomplexes (Gómez and Hagen 2012). While the present study has a limitation in quantifying each complex component expression in adipocytes at the protein level, aging might lead to the translation of OXPHOS complex component proteins at lower efficiency. Interestingly, a whole‐organism study of aging dynamics in mice also defined that both mitochondrial function and inflammatory response were important pathways differentially expressed in aging (Schaum et al. 2020). We thus speculate that COX7RP is a critical factor that drives mitochondrial respiratory supercomplex assembly facilitating efficient electron transfer even in the inner mitochondrial membrane environment with decreasing fluidity. The activation of mitochondrial respiratory supercomplex function via COX7RP may guide the future development of senotherapies.

Among the DEGs abundantly expressed in old WT adipocytes versus old *COX7RP*‐Tg adipocytes, we identified smooth muscle cell markers *Acta2* and *Mylk* as well as preadipocyte marker *Akr1b7*. Because perivascular adipocyte progenitor cells can arise from perivascular mural cells (Lee et al. 2022), we consider that our adipocyte transcriptomic results may show a higher cell transition status from vascular cells into preadipocytes during the aging process. In contrast, *Plin4*, a gene of perilipin that coats the surface of lipid droplets, was downregulated in old *COX7RP*‐Tg adipocytes versus old WT adipocytes. Among perilipin proteins, PLIN4 is most highly associated with skeletal muscle and whose expression can be downregulated in long‐term combined endurance and strength training (Pourteymour et al. 2015). Because PLIN4 is localized to the periphery of muscle fibers and the intramuscular adipocytes (Pourteymour et al. 2015), *Plin4* downregulation in old *COX7RP*‐Tg adipocytes may indicate that COX7RP overexpression can mimic long‐term endurance training that facilitates the cell transition of adipocytes to muscle fibers.

Consistent with our findings, a link between reduced WAT and extended lifespan has been observed in mice with calorie restriction, which reduces circulating insulin levels, reduces WAT mass, and improves mitochondrial function, resulting in decreased oxidative stress and increased lifespan (Bluher 2008). Because insulin resistance can be reversed by the removal of age‐related accumulated visceral fat in rats (Gabriely et al. 2002), calorie restriction is recognized as a promising strategy that prevents senescence and prolongs lifespan in various species (Shimokawa et al. 2008).

We recently developed a FRET‐based screening system to identify regulators of mitochondrial respiratory supercomplex assembly in myoblastic cells (Kobayashi et al. 2023). This screen revealed that spleen tyrosine kinase (SYK) inhibitors play a key role in promoting mitochondrial respiratory supercomplex formation and enhancing respiration. Moreover, SYK inhibition improved muscle performance in mice, highlighting its potential as a therapeutic target. These findings suggest that modulating mitochondrial respiratory supercomplexes may improve physiological function. We thus expect that the identification of activators for COX7RP or mitochondrial respiratory supercomplex assembly, will be potentially relevant to health maintenance and lifespan extension. Modification of mitochondrial respiratory supercomplex assembly or COX7RP functions would be promising for future development of interventions that can prolong longevity or healthy lifespan.

## 4 Materials and Methods

### 4.1 COX7RP‐Tg Mice

All animal experiments were approved by the Animal Care and Use Committee of Saitama Medical University, and conducted in accordance with the Institutional Guidelines and Regulations for the Care and Use of Experimental Animals. Mice were maintained in a temperature‐controlled room (23°C) with a 12‐h light/dark schedule and fed a standard diet (ce2, CLEA Japan), with free access to water. *COX7RP*‐Tg mice expressing Flag‐tagged human COX7RP under CAG (CMV‐IE enhancer, chicken β‐actin promoter, rabbit β‐globin genomic DNA) promoter were generated using a microinjection of fertilized eggs from C57BL/6 mice as described previously (Ikeda et al. 2013).

### 4.2 Measurement of Serum TG, TC, and NEFA Levels

Serum TG, TC, and NEFA levels were measured using the Wako TG E test kit, TC E test kit, and NEFA C test kit, respectively (Wako Pure Chemical).

### 4.3 OGTT

Male *COX7RP*‐Tg and WT mice at 8 months old (*n* = 6 for each group) were fasted for 16 h before OGTT as described (Shiba et al. 2014). Mice were challenged with orally administered d‐(+)‐glucose (2 g kg^−1^). Blood samples were collected from the saphenous vein at 0, 30, 60, and 120 min after glucose administration. Blood glucose levels were measured using a glucose analyzer (Sanwa Kagaku Kenkyusho). Plasma insulin levels were measured using Mouse Insulin ELISA KIT (S‐type) (Shibayagi).

### 4.4 ITT

Male *COX7RP*‐Tg and WT mice at 8 months old (*n* = 6 for each group) were fasted for 1 h before ITT as described (Shiba et al. 2014). Mice were injected intraperitoneally with insulin (0.5 U kg^−1^). Blood samples were collected from the saphenous vein at 0, 30, 60, and 120 min after injection of insulin, and blood glucose levels were measured.

### 4.5 PTT

Male *COX7RP*‐Tg and WT mice at 8 months old (*n* = 5 for each group) were fasted for 16 h before PTT as described (Shiba et al. 2017). Mice were injected intraperitoneally with sodium pyruvate (2 g per kg^−1^). Blood samples were collected from the saphenous vein at 0, 10, 30, 60, 90, and 120 min after injection of pyruvate, and blood glucose levels were measured.

### 4.6 OCR and ECAR

WAT and quadriceps femoris muscle were dissected from male *COX7RP*‐Tg and WT mice at 2 years old (*n* = 6 for each group). The freshly isolated tissues were cut into sections, and a 1 mg piece was placed in each well of XF 24 Islet Capture Microplate (Seahorse Bioscience). The OCR and ECAR were measured using Seahorse XF24 analyzer (Seahorse Bioscience) with Cell Mito Stress Test Kit (Seahorse Bioscience) in which oligomycin (60 μM), FCCP (10 μM), and antimycin A (15 μM) and rotenone (5 μM) were sequentially injected. Basal and maximal OCR were calculated by subtracting the OCR in the presence of antimycin A/rotenone from those before the injection of oligomycin and in the presence of FCCP, respectively.

### 4.7 Mitochondrial ATP Synthesis

ATP synthesis rate was quantified using mitochondria isolated from WAT and quadriceps femoris muscle in 2‐year‐old *COX7RP*‐Tg and WT mice as described previously (Ikeda et al. 2013). Briefly, mitochondria were resuspended in a buffer containing 25 mM Tris–HCl, pH 7.4, 150 mM KCl, 2 mM EDTA, 10 mM potassium phosphate, 0.1 mM MgCl_2_, 0.1% bovine serum albumin, and 50 mg mL^−1^ digitonin, and then energized with 1 mM malate and 1 mM pyruvate in the presence of 1 mM ADP and 0.15 mM adenylate kinase inhibitor P1, P5‐di (adenosine‐50) pentaphosphate for 10 min at 37°C. Parallel incubations were also carried out in the presence of 2 mg mL^−1^ oligomycin to measure mitochondria‐specific ATP synthesis. ATP concentrations were measured using the MicroLumat Plus luminometer (Berthold Technologies) with ATP luminescent reagent (TOYO B‐Net).

### 4.8 ROS Production

ROS production was quantified in WAT and quadriceps femoris muscle, both of which were obtained from 2‐year‐old *COX7RP*‐Tg and WT male mice. Mitochondria were purified from these tissues by homogenization in a buffer (20 mM Tris–HCl, pH 7.4, 150 mM KCl, 0.5 mM EDTA, 1 mM MgCl_2_, 5 mM glucose, and 0.5 mM octanoic acid) followed by centrifugation at 10,000 *g* for 5 min. ROS production in mitochondria was measured using the OxiSelect In Vitro ROS/RNS Assay Kit (CELL BIOLABS) based on a dichlorofluorescin probe.

### 4.9 BN‐PAGE

BN‐PAGE and Western blot analysis of mitochondria extracted from WAT and quadriceps femoris muscle were performed as previously described (Ikeda et al. 2013, 2019). Briefly, tissues were homogenized with a glass‐teflon homogenizer in a buffer containing 10 mM HEPES‐KOH (pH 7.4), 0.22 M mannitol, 0.07 M sucrose, and 0.1 mM EDTA and the mitochondrial fraction was obtained by differential centrifugation. Mitochondria were resuspended in 10 μL of a buffer containing 50 mM Bis‐Tris and 1 M 6‐aminocaproic acid, and subsequently solubilized with digitonin (digitonin/protein ratio of 4 g g^−1^). The solubilized proteins were supplemented with 1 μL of sample buffer (5% Coomassie Brilliant Blue G‐250 in 0.5 M 6‐aminocaproic acid) and separated on BN‐PAGE. Western blotting was performed according to standard protocols (Ikeda et al. 2013) and the blots were probed with anti‐COX1 (1:5000 dilution, ab14705, Abcam) and Fp70 (1:5000 dilution, 459200, Invitrogen).

### 4.10 NAD ^+^ Quantification

The amount of NAD^+^ was quantified using the NAD/NADH Assay Kit‐WST (Dojindo Laboratories) according to the manufacturer's instructions. Briefly, WAT and quadriceps femoris muscle tissues were homogenized in the NAD/NADH extraction buffer and subjected to the enzymatic reaction at 37°C for 1 h. Then, the absorbance of the samples was read on a microplate reader at a wavelength of 450 nm. The NAD^+^ amount was normalized with the protein concentrations.

### 4.11 SA‐β‐Gal

SA‐β‐gal activity was measured using the Senescence β‐galactosidase Activity Assay Kit (Cell Signaling Technology) according to the manufacturer's instructions. Briefly, WAT and gastrocnemius muscle tissues were homogenized in Senescence Cell Lysis Buffer and, then, the cytosolic fractions were plated for fluorescent reading with excitation at 355 nm and emission at 460 nm. For whole tissue staining, freshly isolated WAT was incubated for 12 h at 37°C in a β‐gal staining solution containing 1 mg mL^−1^ 5‐bromo‐4‐chloro‐3‐indolyl β‐D‐galactopyranoside (X‐gal), 5 mmol L^−1^ potassium ferrocyanide, 5 mmol L^−1^ potassium fericyanide, 150 mmol L^−1^ NaCl, 2 mmol L^−1^ MgCl_2_, 0.01% sodium deoxycholate, and 0.02% Nonidet‐40.

### 4.12 Western Blot Analysis

Tissues were prepared in sample buffer for sodium dodecyl sulfate–polyacrylamide gel electrophoresis (SDS‐PAGE), heated at 100°C for 15 min, and subjected to SDS‐PAGE followed by western blot analysis using antibodies against Cox7rp (1:1000 dilution), Risp (1:5000 dilution, ab14746, Abcam), Cox1 (1:5000 dilution, ab14705, Abcam), Ndufa9 (1:5000 dilution, 459100, Invitrogen), and β‐Actin (1:4000 dilution, AC‐74, Sigma‐Aldrich). The signal intensity of each protein band was quantified densitometrically using ImageJ software (https://imagej.nih.gov/ij/). The densitometric value of each protein was normalized to the corresponding β‐Actin signal and presented as a relative value. Mouse monoclonal anti‐COX7RP antibody was generated using a polypeptide containing amino acids 101–114 of the human COX7RP protein as described elsewhere (Sato et al. 2021).

### 4.13 qRT‐PCR

Total RNAs were extracted from the tissues in *COX7RP*‐Tg and WT mice using ISOGEN reagent (Nippon Gene) and then subjected to cDNA synthesis using reverse transcriptase SuperScript II (Invitrogen). qPCR was performed with StepOnePlus (Applied Biosystems) using KAPA SYBR Fast qPCR Kit (Nippon Genetics) to determine the expression levels of human and mouse *COX7RP*, *Ccl5*, and *Tnf*. The relative expression levels of genes were calculated by normalization with *Gapdh* expression as an internal control. The sequences of PCR primers are as follows: common primers for human and mouse COX7RP forward, 5′‐GTTTCCACAGAAGCACCACCTATC‐3′; human and mouse COX7RP reverse, *Ccl5* forward, 5′‐CCCTCACCATCATCCTCACTG‐3′; *Ccl5* reverse, 5′‐GAGAGGTAGGCAAAGCAGCAG‐3′; *Tnf* forward, 5′‐ACCGTCAGCCGATTTGCTAT‐3′; *Tnf* reverse, 5′‐CTTGACGGCAGAGAGGAGGTT‐3′; *Gapdh* forward, 5′‐GCATGGCCTTCCGTGTTC‐3′; and *Gapdh* reverse, 5′‐TGTCATCATACTTGGCAGGTTTCT‐3′.

### 4.14 snRNA‐Seq Library Preparation and Sequencing

Single‐cell suspensions were prepared from the WAT of *COX7RP*‐Tg and WT mice at 8 weeks old and 2 years old. Single cell nuclei were isolated using Rosen's protocol (Emont et al. 2022). Each flash‐frozen adipose tissue sample was placed into a gentleMACS C tube (Miltenyi Biotec) with 2 mL freshly prepared TST buffer (0.03% Tween 20 [Bio‐Rad], 0.01% Molecular Grade BSA [New England Biolabs], 146 mM NaCl [ThermoFisher Scientific], 1 mM CaCl_2_ [VWR International], 21 mM MgCl_2_ [Sigma Aldrich], and 10 mM Tris–HCl pH 7.5 [ThermoFisher Scientific] in Ultrapure water [ThermoFisher Scientific]) with or without 0.2 U μL^−1^ of Protector RNase Inhibitor (Sigma Aldrich). gentleMACS C tubes were then placed on the gentleMACS Dissociator (Miltenyi Biotec) and tissue was dissociated by running the program mr_adipose_01 twice, and then incubated on ice for 10 min. Lysate was passed through a 40‐μm nylon filter (CellTreat) and collected into a 50‐mL conical tube (Corning). The filter was rinsed with 3 mL of freshly prepared ST buffer (146 mM NaCl, 1 mM CaCl_2_, 21 mM MgCl_2_; 10 mM Tris–HCl pH 7.5) with or without 0.2 U μL^−1^ RNase Inhibitor, and collected into the same tube. Flow‐through was centrifuged at 500 *g* for 5 min at 4°C with the brake set to low. Following centrifugation, the supernatant was removed, and the nuclear pellet was resuspended in 50–200 μL PBS pH 7.4 (ThermoFisher Scientific) with 0.02% BSA, with or without 0.2 U μL^−1^ RNase inhibitor. Nuclei from each sample were stained with ViaStain AO/PI Staining Solution (Nexcelom Biosciences) and counted in a hemocytometer using fluorescence to identify intact nuclei. The snRNA‐seq libraries were prepared with a Fixed RNA profiling reagent kit according to 10× Genomics specifications. After hybridization, nuclei from each sample were stained with ViaStain AO/PI Staining Solution (Nexcelom Biosciences) and counted in a hemocytometer again. For each sample, bc001, bc002, bc003, and bc004 barcodes were added. Samples were pooled to achieve an expected cell count of 10,000 cells each (10× Genomics), then loaded in one channel of a Chromium Chip (10× Genomics). The indexed libraries were subjected to a second round of double‐sided size selection, and the libraries were then quantified and quality‐assessed with Agilent TapeStation. The libraries were clustered using NovaSeq 6000 on a paired‐end read flow cell. Sequencing was performed with a 100 cycle kit and the following read structure: Read1: 28, index1(i7): 10, index2(i5): 10, Read2: 90. The 10× Genomics Cell Ranger Single Cell Software was used for sample demultiplexing, alignment to the mouse reference genome (mm10), filtering, UMI counting, single‐cell 3′‐end gene counting, and quality control according to the manufacturer's parameters.

### 4.15 snRNA‐Seq Data Analysis

The R package Seurat (v4.0.1) was used to cluster cells in a merged matrix. Several quality control steps were performed based on the plot pattern of the expression gene number and ratio of mitochondrial RNA in each sample. First, cells expressing fewer than 200 genes or > 5% mitochondrial genes (low quality) were excluded from further analysis. The gene counts for each cell were divided by the total gene counts for each cell and multiplied by a scale factor of 10,000, after which a natural log transformation was applied. The FindVariableFeatures function was used to select variable genes with default parameters. The ScaleData function was used to scale and center the counts in the dataset. To define the cell types of all cells, we performed principal component analysis on the variable genes, and 15 principal components were used for cell clustering (resolution = 0.5) and UMAP dimensional reduction. The cluster markers were found using the FindAllMarkers function, and cell types were manually annotated based on the cluster markers. DEGs between two groups were defined by FindMarkers with Welch *t*‐test. The single‐cell gene expression data were visualized with UMAP overlays, dot heatmaps, and violin plots. A volcano plot was constructed in R. Gene Ontology analysis was performed using enrichGO in clusterProfiler. All of the subontologies (Molecular Function, Biological Process, and Cellular Component) were included in the list, and the method for adjusting the *p* value was Benjamini and Hochberg.

### 4.16 SASP Gene Scoring

We selected major SASP‐associated genes based on the gene expression information in the WAT snRNA‐seq (Emont et al. 2022) and a list of SASP‐related genes (Freund et al. 2010). The list included *Il6*, *Cxcl1*, *Il1b*, *Tnf*, *Tgfb1*, *Egf*, *Mmp3*, *Mmp9*, *Igfbp5*, *Adam17*, *Ctsb*, *Ctsl*, *Ctsd*, *Il18*, *Ccl2*, *Ccl3*, *Ccl5*, and *Ccl6*. To calculate the overall SASP induction, the R package of calculate_signature_scores was used. The result calculated from the sum of *Z*‐scores within SASP‐associated genes was presented as the gene.score. A significance test was conducted using the one‐way ANOVA followed by the Tukey method, and *p* values were calculated for each group comparison.

### 4.17 Identification of TFs by SCENIC

SCENIC was used to identify the gene regulatory networks (GRNs) and the upstream TF from snRNA‐seq data in R (Aibar et al. 2017). To identify TF binding motifs, the database (mm10_refseq‐r80_10kb_up_and_down_tss.mc9nr.feather/mm10_refseq‐r80_500bp_up_ and_100bp_down_tss.mc9nr.feather) was downloaded as cisTarget_database in the RcisTarget package. For running GENIE3, the parameter of ntree in the random forest model was 500. Among the scored GRNs, the extended regulons were selected as predicted upstream TFs. TF identification was performed in two pairs of comparisons: young WT (8‐week‐old) versus old WT (2‐year‐old), and old WT versus old *COX7RP*‐Tg. The regulon specificity score (RSS) was calculated by the calcRSS package and plotted by the plotRSS package with *Z* score normalization (Suo et al. 2018) across the samples between the pairs, individually.

### 4.18 Statistical Analysis

Significance of differences between two groups was analyzed by a two‐tailed unpaired Student's *t*‐test. The details of statistical analysis in snRNA‐seq data analysis, SASP gene scoring, and SCRNIC analysis were described above.

## Author Contributions

K.I.: conceptualization; data curation; investigation; funding acquisition; writing – original draft. S.S.: conceptualization; data curation; investigation; funding acquisition; writing – original draft. M.Y.: data curation; investigation; writing – original draft. M.F.: data curation; investigation; writing – original draft. K.H.: formal analysis; validation; funding acquisition; writing – review and editing. T.T.: methodology; supervision; writing – review and editing. S.I.: conceptualization; funding acquisition; supervision; writing – review and editing.

## Conflicts of Interest

The authors declare no conflicts of interest.

## Supporting information


**Figure S1:** acel70294‐sup‐0001‐FigureS1.pdf.

**Figure S2:** acel70294‐sup‐0002‐FigureS2.pdf.

**Figure S3:** acel70294‐sup‐0003‐FigureS3.pdf.

**Figure S4:** acel70294‐sup‐0004‐FigureS4.pdf.

**Figure S5:** acel70294‐sup‐0005‐FigureS5.pdf.

**Figure S6:** acel70294‐sup‐0006‐FigureS6.pdf.

**Figure S7:** acel70294‐sup‐0007‐FigureS7.pdf.

**Figure S8:** acel70294‐sup‐0008‐FigureS8.pdf.

**Figure S9:** acel70294‐sup‐0009‐FigureS9.pdf.

## Data Availability

All data needed to evaluate the conclusions in the paper are present in the paper and/or the Supporting Information. The snRNA‐seq data have been deposited with links to BioProject accession number PRJDB17809 in the DDBJ BioProject database.

## References

[acel70294-bib-0001] Aibar, S. , C. B. González‐Blas , T. Moerman , et al. 2017. “SCENIC: Single‐Cell Regulatory Network Inference and Clustering.” Nature Methods 14, no. 11: 1083–1086. 10.1038/nmeth.4463. 28991892 PMC5937676

[acel70294-bib-0002] Balaban, R. S. , S. Nemoto , and T. Finkel . 2005. “Mitochondria, Oxidants, and Aging.” Cell 120, no. 4: 483–495. 10.1016/j.cell.2005.02.001. 15734681

[acel70294-bib-0003] Benegiamo, G. , M. Bou Sleiman , M. Wohlwend , et al. 2022. “COX7A2L Genetic Variants Determine Cardiorespiratory Fitness in Mice and Human.” Nature Metabolism 4, no. 10: 1336–1351. 10.1038/s42255-022-00655-0. PMC9584823 36253618

[acel70294-bib-0004] Bennett, C. F. , P. Latorre‐Muro , and P. Puigserver . 2022. “Mechanisms of Mitochondrial Respiratory Adaptation.” Nature Reviews. Molecular Cell Biology 23, no. 12: 817–835. 10.1038/s41580-022-00506-6. 35804199 PMC9926497

[acel70294-bib-0005] Bluher, M. 2008. “Fat Tissue and Long Life.” Obesity Facts 1, no. 4: 176–182. 10.1159/000145930. 20054178 PMC6452107

[acel70294-bib-0006] Bou Sleiman, M. , P. Jha , R. Houtkooper , R. W. Williams , X. Wang , and J. Auwerx . 2020. “The Gene‐Regulatory Footprint of Aging Highlights Conserved Central Regulators.” Cell Reports 32, no. 13: 108203. 10.1016/j.celrep.2020.108203. 32997995 PMC7527782

[acel70294-bib-0007] Chandra, P. K. , S. Cikic , I. Rutkai , et al. 2022. “Effects of Aging on Protein Expression in Mice Brain Microvessels: ROS Scavengers, mRNA/Protein Stability, Glycolytic Enzymes, Mitochondrial Complexes, and Basement Membrane Components.” Geroscience 44, no. 1: 371–388. 10.1007/s11357-021-00468-1. 34708300 PMC8811117

[acel70294-bib-0008] Cogliati, S. , E. Calvo , M. Loureiro , et al. 2016. “Mechanism of Super‐Assembly of Respiratory Complexes III and IV.” Nature 539, no. 7630: 579–582. 10.1038/nature20157. 27775717

[acel70294-bib-0009] Cornier, M.‐A. , D. Dabelea , T. L. Hernandez , et al. 2008. “The Metabolic Syndrome.” Endocrine Reviews 29, no. 7: 777–822. 10.1210/er.2008-0024. 18971485 PMC5393149

[acel70294-bib-0010] Deng, W.‐G. , Y. Zhu , and K. K. Wu . 2004. “Role of p300 and PCAF in Regulating Cyclooxygenase‐2 Promoter Activation by Inflammatory Mediators.” Blood 103, no. 6: 2135–2142. 10.1182/blood-2003-09-3131. 14630807

[acel70294-bib-0011] Emont, M. P. , C. Jacobs , A. L. Essene , et al. 2022. “A Single‐Cell Atlas of Human and Mouse White Adipose Tissue.” Nature 603, no. 7903: 926–933. 10.1038/s41586-022-04518-2. 35296864 PMC9504827

[acel70294-bib-0012] Frenzel, M. , H. Rommelspacher , M. D. Sugawa , and N. A. Dencher . 2010. “Ageing Alters the Supramolecular Architecture of OxPhos Complexes in Rat Brain Cortex.” Experimental Gerontology 45, no. 7–8: 563–572. 10.1016/j.exger.2010.02.003. 20159033

[acel70294-bib-0013] Freund, A. , A. V. Orjalo , P.‐Y. Desprez , and J. Campisi . 2010. “Inflammatory Networks During Cellular Senescence: Causes and Consequences.” Trends in Molecular Medicine 16, no. 5: 238–246. 10.1016/j.molmed.2010.03.003. 20444648 PMC2879478

[acel70294-bib-0014] Gabriely, I. , X. H. Ma , X. M. Yang , et al. 2002. “Removal of Visceral Fat Prevents Insulin Resistance and Glucose Intolerance of Aging: An Adipokine‐Mediated Process?” Diabetes 51, no. 10: 2951–2958. 10.2337/diabetes.51.10.2951. 12351432

[acel70294-bib-0015] Goates, S. , K. Du , M. B. Arensberg , T. Gaillard , J. Guralnik , and S. L. Pereira . 2019. “Economic Impact of Hospitalizations in US Adults With Sarcopenia.” Journal of Frailty & Aging 8, no. 2: 93–99. 10.14283/jfa.2019.10. 30997923 PMC12275775

[acel70294-bib-0016] Gómez, L. A. , and T. M. Hagen . 2012. “Age‐Related Decline in Mitochondrial Bioenergetics: Does Supercomplex Destabilization Determine Lower Oxidative Capacity and Higher Superoxide Production?” Seminars in Cell & Developmental Biology 23, no. 7: 758–767. 10.1016/j.semcdb.2012.04.002. 22521482 PMC4096948

[acel70294-bib-0017] Han, H. , J.‐W. Cho , S. Lee , et al. 2018. “TRRUST v2: An Expanded Reference Database of Human and Mouse Transcriptional Regulatory Interactions.” Nucleic Acids Research 46, no. D1: D380–D386. 10.1093/nar/gkx1013. 29087512 PMC5753191

[acel70294-bib-0018] Hasegawa, Y. , T. Saito , T. Ogihara , et al. 2012. “Blockade of the Nuclear Factor‐κB Pathway in the Endothelium Prevents Insulin Resistance and Prolongs Life Spans.” Circulation 125, no. 9: 1122–1133. 10.1161/CIRCULATIONAHA.111.054346. 22302838

[acel70294-bib-0019] Ikeda, K. , K. Horie‐Inoue , T. Suzuki , et al. 2019. “Mitochondrial Supercomplex Assembly Promotes Breast and Endometrial Tumorigenesis by Metabolic Alterations and Enhanced Hypoxia Tolerance.” Nature Communications 10, no. 1: 4108. 10.1038/s41467-019-12124-6. PMC6739376 31511525

[acel70294-bib-0020] Ikeda, K. , S. Shiba , K. Horie‐Inoue , K. Shimokata , and S. Inoue . 2013. “A Stabilizing Factor for Mitochondrial Respiratory Supercomplex Assembly Regulates Energy Metabolism in Muscle.” Nature Communications 4: 2147. 10.1038/ncomms3147. 23857330

[acel70294-bib-0021] Johnson, C. , R. Williams , J. Y. Wei , and G. Ranganathan . 2011. “Regulation of Serum Response Factor and Adiponectin by PPARγ Agonist Docosahexaenoic Acid.” Journal of Lipids 2011: 670479. 10.1155/2011/670479. 21490806 PMC3066850

[acel70294-bib-0022] Kauppila, T. E. S. , J. H. K. Kauppila , and N.‐G. Larsson . 2017. “Mammalian Mitochondria and Aging: An Update.” Cell Metabolism 25, no. 1: 57–71. 10.1016/j.cmet.2016.09.017. 28094012

[acel70294-bib-0023] Kennedy, B. K. , S. L. Berger , A. Brunet , et al. 2014. “Geroscience: Linking Aging to Chronic Disease.” Cell 159, no. 4: 709–713. 10.1016/j.cell.2014.10.039. 25417146 PMC4852871

[acel70294-bib-0024] Kobayashi, A. , K. Azuma , T. Takeiwa , et al. 2023. “A FRET‐Based Respirasome Assembly Screen Identifies Spleen Tyrosine Kinase as a Target to Improve Muscle Mitochondrial Respiration and Exercise Performance in Mice.” Nature Communications 14, no. 1: 312. 10.1038/s41467-023-35865-x. PMC9877034 36697396

[acel70294-bib-0025] Lapuente‐Brun, E. , R. Moreno‐Loshuertos , R. Acín‐Pérez , et al. 2013. “Supercomplex Assembly Determines Electron Flux in the Mitochondrial Electron Transport Chain.” Science 340, no. 6140: 1567–1570. 10.1126/science.1230381. 23812712

[acel70294-bib-0026] Lee, S. , A. M. Benvie , H. G. Park , et al. 2022. “Remodeling of Gene Regulatory Networks Underlying Thermogenic Stimuli‐Induced Adipose Beiging.” Communications Biology 5, no. 1: 584. 10.1038/s42003-022-03531-5. 35701601 PMC9197980

[acel70294-bib-0027] Lobo‐Jarne, T. , E. Nývltová , R. Pérez‐Pérez , et al. 2018. “Human COX7A2L Regulates Complex III Biogenesis and Promotes Supercomplex Organization Remodeling Without Affecting Mitochondrial Bioenergetics.” Cell Reports 25, no. 7: 1786–1799.e4. 10.1016/j.celrep.2018.10.058. 30428348 PMC6286155

[acel70294-bib-0028] Lombardi, A. , E. Silvestri , F. Cioffi , et al. 2009. “Defining the Transcriptomic and Proteomic Profiles of Rat Ageing Skeletal Muscle by the Use of a cDNA Array, 2D‐ and Blue Native‐PAGE Approach.” Journal of Proteomics 72, no. 4: 708–721. 10.1016/j.jprot.2009.02.007. 19268720

[acel70294-bib-0029] Longobucco, Y. , C. Benedetti , S. Tagliaferri , et al. 2019. “Proactive Interception and Care of Frailty and Multimorbidity in Older Persons: The Experience of the European Innovation Partnership on Active and Healthy Ageing and the Response of Parma Local Health Trust and Lab Through European Projects.” Acta Biomed 90, no. 2: 364–374. 10.23750/abm.v90i2.8419. 31125023 PMC6776195

[acel70294-bib-0030] Lopes‐Paciencia, S. , E. Saint‐Germain , M.‐C. Rowell , A. F. Ruiz , P. Kalegari , and G. Ferbeyre . 2019. “The Senescence‐Associated Secretory Phenotype and Its Regulation.” Cytokine 117: 15–22. 10.1016/j.cyto.2019.01.013. 30776684

[acel70294-bib-0031] Lopez‐Fabuel, I. , J. Le Douce , A. Logan , et al. 2016. “Complex I Assembly Into Supercomplexes Determines Differential Mitochondrial ROS Production in Neurons and Astrocytes.” Proceedings of the National Academy of Sciences of the United States of America 113, no. 46: 13063–13068. 10.1073/pnas.1613701113. 27799543 PMC5135366

[acel70294-bib-0032] Maniyadath, B. , Q. Zhang , R. K. Gupta , and S. Mandrup . 2023. “Adipose Tissue at Single‐Cell Resolution.” Cell Metabolism 35, no. 3: 386–413. 10.1016/j.cmet.2023.02.002. 36889280 PMC10027403

[acel70294-bib-0033] Minamino, T. , M. Orimo , I. Shimizu , et al. 2009. “A Crucial Role for Adipose Tissue p53 in the Regulation of Insulin Resistance.” Nature Medicine 15, no. 9: 1082–1087. 10.1038/nm.2014. 19718037

[acel70294-bib-0034] Müller‐Höcker, J. 1989. “Cytochrome‐c‐Oxidase Deficient Cardiomyocytes in the Human Heart—An Age‐Related Phenomenon. A Histochemical Ultracytochemical Study.” American Journal of Pathology 134, no. 5: 1167–1173. 2541614 PMC1879907

[acel70294-bib-0035] Murase, R. , A. Yamamoto , Y. Hirata , and K. Oh‐Hashi . 2022. “Expression Analysis and Functional Characterization of Thioredoxin Domain‐Containing Protein 11.” Molecular Biology Reports 49, no. 11: 10541–10556. 10.1007/s11033-022-07932-x. 36152228

[acel70294-bib-0036] Nelson, G. , O. Kucheryavenko , J. Wordsworth , and T. von Zglinicki . 2018. “The Senescent Bystander Effect Is Caused by ROS‐Activated NF‐κB Signalling.” Mechanisms of Ageing and Development 170: 30–36. 10.1016/j.mad.2017.08.005. 28837845 PMC5861994

[acel70294-bib-0037] Novack, G. V. , P. Galeano , E. M. Castaño , and L. Morelli . 2020. “Mitochondrial Supercomplexes: Physiological Organization and Dysregulation in Age‐Related Neurodegenerative Disorders.” Frontiers in Endocrinology 11: 600. 10.3389/fendo.2020.00600. 33042002 PMC7518391

[acel70294-bib-0038] Perez‐Campo, R. , M. López‐Torres , S. Cadenas , C. Rojas , and G. Barja . 1998. “The Rate of Free Radical Production as a Determinant of the Rate of Aging: Evidence From the Comparative Approach.” Journal of Comparative Physiology B Biochemical, Systemic, and Environmental Physiology 168, no. 3: 149–158. 10.1007/s003600050131. 9591361

[acel70294-bib-0039] Pérez‐Pérez, R. , T. Lobo‐Jarne , D. Milenkovic , et al. 2016. “COX7A2L Is a Mitochondrial Complex III Binding Protein That Stabilizes the III2+IV Supercomplex Without Affecting Respirasome Formation.” Cell Reports 16, no. 9: 2387–2398. 10.1016/j.celrep.2016.07.081. 27545886 PMC5007171

[acel70294-bib-0040] Pourteymour, S. , S. Lee , T. M. Langleite , et al. 2015. “Perilipin 4 in Human Skeletal Muscle: Localization and Effect of Physical Activity.” Physiological Reports 3, no. 8: e12481. 10.14814/phy2.12481. 26265748 PMC4562567

[acel70294-bib-0041] Radovic, M. , L. P. Gartzke , S. E. Wink , J. A. van der Kleij , F. A. Politiek , and G. Krenning . 2025. “Targeting the Electron Transport System for Enhanced Longevity.” Biomolecules 15, no. 5: 614. 10.3390/biom15050614. 40427507 PMC12109555

[acel70294-bib-0042] Rovira, M. , R. Sereda , D. Pladevall‐Morera , et al. 2022. “The Lysosomal Proteome of Senescent Cells Contributes to the Senescence Secretome.” Aging Cell 21, no. 10: e13707. 10.1111/acel.13707. 36087066 PMC9577959

[acel70294-bib-0043] Roy, D. , K. T. Farabaugh , J. Wu , et al. 2017. “Coordinated Transcriptional Control of Adipocyte Triglyceride Lipase (Atgl) by Transcription Factors Sp1 and Peroxisome Proliferator‐Activated Receptor γ (PPARγ) During Adipocyte Differentiation.” Journal of Biological Chemistry 292, no. 36: 14827–14835. 10.1074/jbc.M117.783043. 28726642 PMC5592664

[acel70294-bib-0044] Sato, J. , K. Azuma , K. Kinowaki , et al. 2021. “Combined Use of Immunoreactivities of RIG‐I With Efp/TRIM25 for Predicting Prognosis of Patients With Estrogen Receptor‐Positive Breast Cancer.” Clinical Breast Cancer 21, no. 5: 399–407.e2. 10.1016/j.clbc.2020.12.001. 33386231

[acel70294-bib-0045] Schaum, N. , B. Lehallier , O. Hahn , et al. 2020. “Ageing Hallmarks Exhibit Organ‐Specific Temporal Signatures.” Nature 583, no. 7817: 596–602. 10.1038/s41586-020-2499-y. 32669715 PMC7757734

[acel70294-bib-0046] Shiba, S. , K. Ikeda , K. Azuma , et al. 2014. “γ‐Glutamyl Carboxylase in Osteoblasts Regulates Glucose Metabolism in Mice.” Biochemical and Biophysical Research Communications 453, no. 3: 350–355. 10.1016/j.bbrc.2014.09.091. 25264202

[acel70294-bib-0047] Shiba, S. , K. Ikeda , K. Horie‐Inoue , A. Nakayama , T. Tanaka , and S. Inoue . 2017. “Deficiency of COX7RP, a Mitochondrial Supercomplex Assembly Promoting Factor, Lowers Blood Glucose Level in Mice.” Scientific Reports 7, no. 1: 7606. 10.1038/s41598-017-08081-z. 28790391 PMC5548899

[acel70294-bib-0048] Shimokawa, I. , T. Chiba , H. Yamaza , and T. Komatsu . 2008. “Longevity Genes: Insights From Calorie Restriction and Genetic Longevity Models.” Molecules and Cells 26, no. 5: 427–435. 18799928

[acel70294-bib-0049] Srivastava, A. , E. Barth , M. A. Ermolaeva , et al. 2020. “Tissue‐Specific Gene Expression Changes Are Associated With Aging in Mice.” Genomics, Proteomics & Bioinformatics 18, no. 4: 430–442. 10.1016/j.gpb.2020.12.001. PMC8242333 33309863

[acel70294-bib-0050] Sun, N. , R. J. Youle , and T. Finkel . 2016. “The Mitochondrial Basis of Aging.” Molecular Cell 61, no. 5: 654–666. 10.1016/j.molcel.2016.01.028. 26942670 PMC4779179

[acel70294-bib-0051] Suo, S. , Q. Zhu , A. Saadatpour , L. Fei , G. Guo , and G.‐C. Yuan . 2018. “Revealing the Critical Regulators of Cell Identity in the Mouse Cell Atlas.” Cell Reports 25, no. 6: 1436–1445.e3. 10.1016/j.celrep.2018.10.045. 30404000 PMC6281296

[acel70294-bib-0052] Swärd, K. , K. G. Stenkula , C. Rippe , A. Alajbegovic , M. F. Gomez , and S. Albinsson . 2016. “Emerging Roles of the Myocardin Family of Proteins in Lipid and Glucose Metabolism.” Journal of Physiology 594, no. 17: 4741–4752. 10.1113/JP271913. 27060572 PMC5009794

[acel70294-bib-0053] Tang, Q. Q. , M. S. Jiang , and M. D. Lane . 1999. “Repressive Effect of Sp1 on the C/EBPalpha Gene Promoter: Role in Adipocyte Differentiation.” Molecular and Cellular Biology 19, no. 7: 4855–4865. 10.1128/MCB.19.7.4855. 10373535 PMC84284

[acel70294-bib-0054] Vercellino, I. , and L. A. Sazanov . 2021. “Structure and Assembly of the Mammalian Mitochondrial Supercomplex CIII2CIV.” Nature 598, no. 7880: 364–367. 10.1038/s41586-021-03927-z. 34616041

[acel70294-bib-0055] Verdin, E. 2015. “NAD^+^ in Aging, Metabolism, and Neurodegeneration.” Science 350, no. 6265: 1208–1213. 10.1126/science.aac4854. 26785480

[acel70294-bib-0056] Wang, D. , P. S. Chang , Z. Wang , et al. 2001. “Activation of Cardiac Gene Expression by Myocardin, a Transcriptional Cofactor for Serum Response Factor.” Cell 105, no. 7: 851–862. 10.1016/s0092-8674(01)00404-4. 11439182

[acel70294-bib-0057] Wilcox, H. G. , and M. Heimberg . 1987. “Secretion and Uptake of Nascent Hepatic Very Low Density Lipoprotein by Perfused Livers From Fed and Fasted Rats.” Journal of Lipid Research 28, no. 4: 351–360. 3585170

[acel70294-bib-0058] Yeung, F. , J. E. Hoberg , C. S. Ramsey , et al. 2004. “Modulation of NF‐kappaB‐Dependent Transcription and Cell Survival by the SIRT1 Deacetylase.” EMBO Journal 23, no. 12: 2369–2380. 10.1038/sj.emboj.7600244. 15152190 PMC423286

[acel70294-bib-0059] Zhang, L. , L. E. Pitcher , M. J. Yousefzadeh , L. J. Niedernhofer , P. D. Robbins , and Y. Zhu . 2022. “Cellular Senescence: A Key Therapeutic Target in Aging and Diseases.” Journal of Clinical Investigation 132, no. 15: e158450. 10.1172/JCI158450. 35912854 PMC9337830

[acel70294-bib-0060] Zhao, D. , Z.‐K. Huang , Y. Liang , et al. 2024. “Monocytes Release Pro‐Cathepsin D to Drive Blood‐to‐Brain Transcytosis in Diabetes.” Circulation Research 134, no. 7: e17–e33. 10.1161/CIRCRESAHA.123.323622. 38420756

[acel70294-bib-0061] Zhou, Y. , B. Zhou , L. Pache , et al. 2019. “Metascape Provides a Biologist‐Oriented Resource for the Analysis of Systems‐Level Datasets.” Nature Communications 10, no. 1: 1523. 10.1038/s41467-019-09234-6. PMC6447622 30944313

