# Supplementary material for: Mitochondrial Respiratory Supercomplex Assembly Factor COX7RP Contributes to Lifespan Extension in Mice
Source: Aging Cell. 2025 Nov 18;25(1):e70294. doi: 10.1111/acel.70294 (PMC12740103; doi:10.1111/acel.70294)
Supplement: Supplementary file 1 — Figure S1: acel70294‐sup‐0001‐FigureS1.pdf. [file ACEL-25-e70294-s007.pdf]

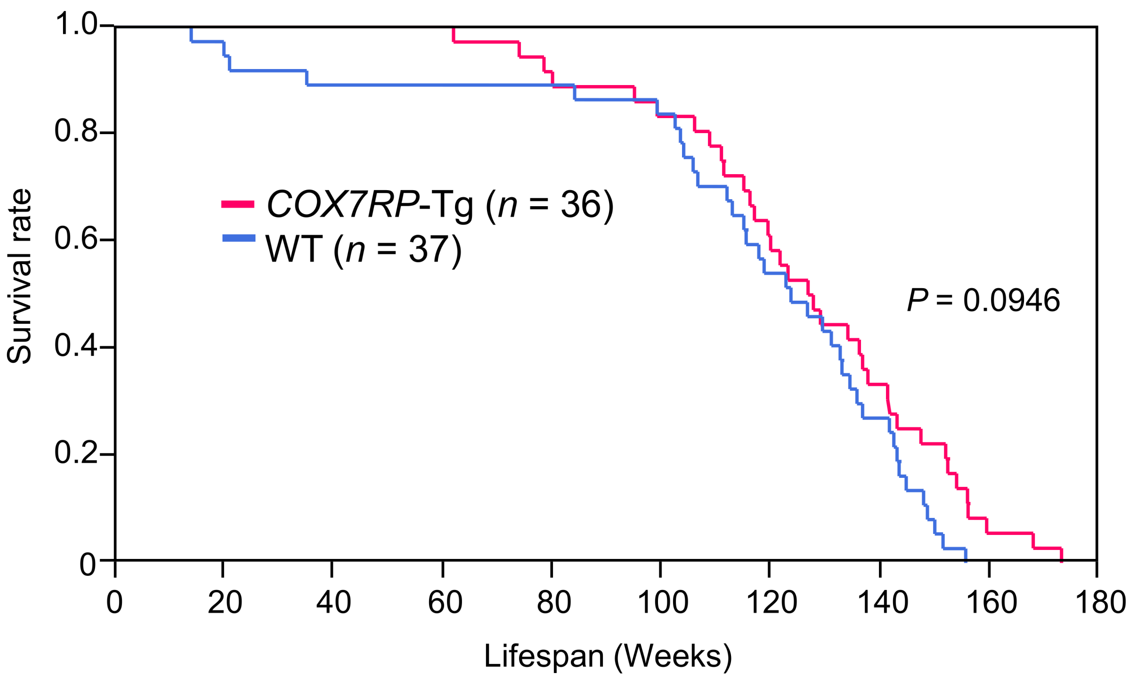

Figure S1 Llifespan of female *COX7RP*-Tg and WT mice. Kaplan-Meier survival curves of *COX7RP*-Tg (n = 36) and control WT littermate (n = 37) mice. Differences between *COX7RP*-Tg and WT mice were analyzed using the log-rank test.
