# Supplementary material for: Mitochondrial Respiratory Supercomplex Assembly Factor COX7RP Contributes to Lifespan Extension in Mice
Source: Aging Cell. 2025 Nov 18;25(1):e70294. doi: 10.1111/acel.70294 (PMC12740103; doi:10.1111/acel.70294)
Supplement: Supplementary file 2 — Figure S2: acel70294‐sup‐0002‐FigureS2.pdf. [file ACEL-25-e70294-s004.pdf]

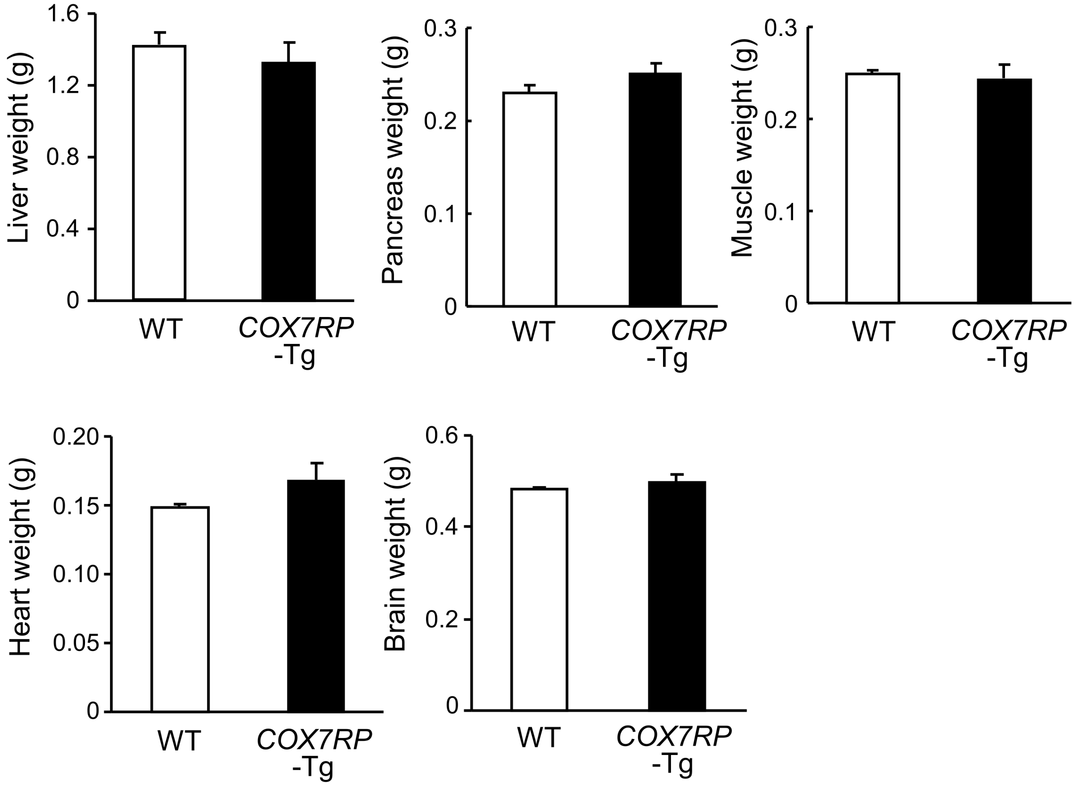

Figure S2 Tissue weights of liver, pancreas, muscle, heart, and brain of male COX7RP-Tg and WT mice at 10 months old. Data are presented as means ± SEM (n = 9). Differences between *COX7RP*-Tg and WT mice were analyzed using a two-tailed Student t-test but did not reach statistical significance.
