# Supplementary material for: Mitochondrial Respiratory Supercomplex Assembly Factor COX7RP Contributes to Lifespan Extension in Mice
Source: Aging Cell. 2025 Nov 18;25(1):e70294. doi: 10.1111/acel.70294 (PMC12740103; doi:10.1111/acel.70294)
Supplement: Supplementary file 3 — Figure S3: acel70294‐sup‐0003‐FigureS3.pdf. [file ACEL-25-e70294-s003.pdf]

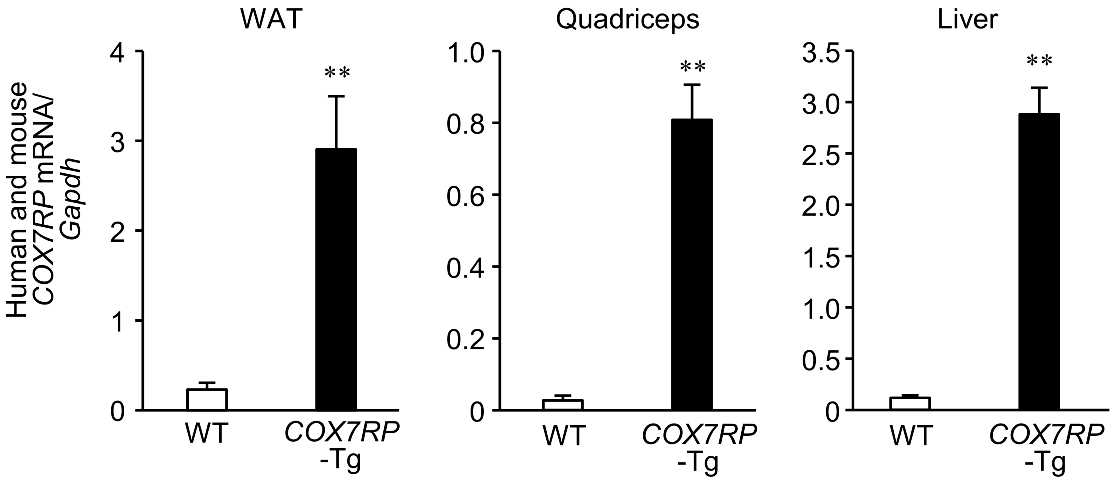

Figure S3 Expression levels of human and mouse *COX7RP* mRNA in WAT, quadriceps femoris muscles, and livers of male *COX7RP*-Tg (n = 5) and WT (n = 5) mice at 8 weeks old. Data are presented as means ± SD (n = 5). Differences between *COX7RP*-Tg and WT mice were analyzed using a two-tailed Student t-test. **$P < 0.01$.
