# Supplementary material for: Mitochondrial Respiratory Supercomplex Assembly Factor COX7RP Contributes to Lifespan Extension in Mice
Source: Aging Cell. 2025 Nov 18;25(1):e70294. doi: 10.1111/acel.70294 (PMC12740103; doi:10.1111/acel.70294)
Supplement: Supplementary file 4 — Figure S4: acel70294‐sup‐0004‐FigureS4.pdf. [file ACEL-25-e70294-s001.pdf]

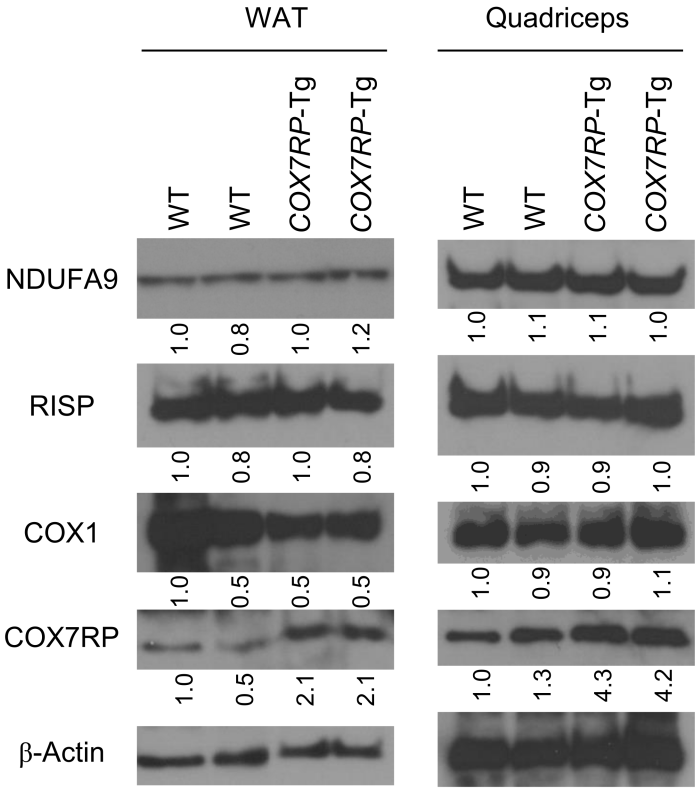

Figure S4 Protein levels of Ndufa9, Risp, Cox1, Cox7rp, and β-Actin in WT and *COX7RP*-Tg mice. Whole cell extracts of WATs and Quadriceps muscles were prepared from 2-year-old WT and *COX7RP*-Tg mice and analyzed by Western blot with specific antibodies for NDUFA9, RISP, COX1, COX7RP, and β-Actin. Blots were quantitated with ImageJ software and the density of signals was normalized to β-Actin.
