# Supplementary material for: Mitochondrial Respiratory Supercomplex Assembly Factor COX7RP Contributes to Lifespan Extension in Mice
Source: Aging Cell. 2025 Nov 18;25(1):e70294. doi: 10.1111/acel.70294 (PMC12740103; doi:10.1111/acel.70294)
Supplement: Supplementary file 5 — Figure S5: acel70294‐sup‐0005‐FigureS5.pdf. [file ACEL-25-e70294-s005.pdf]

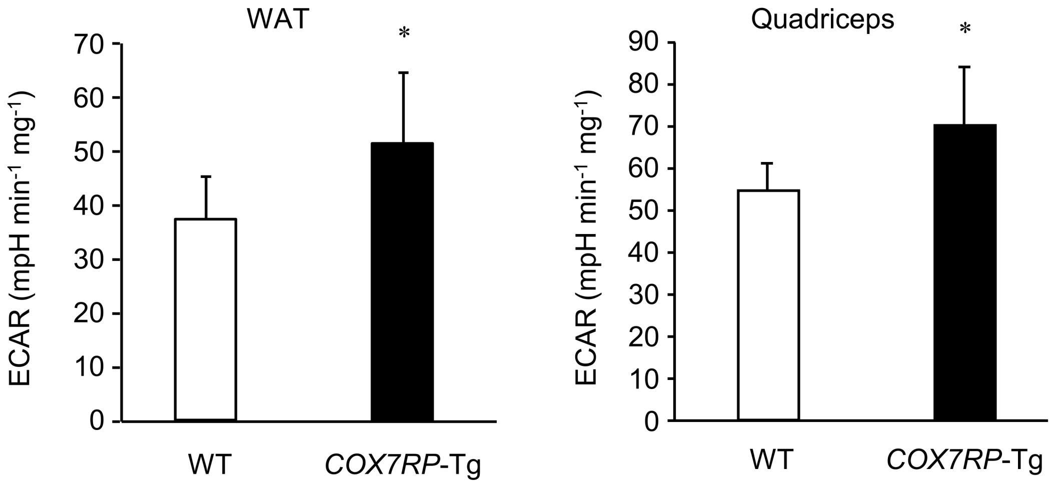

Figure S5 Elevated ECAR in male *COX7RP*-Tg mice. Basal ECAR was measured in quadriceps femoris muscles and WATs dissected from 2-year-old *COX7RP*-Tg and WT mice using Flux analyzer. Data are presented as means ± SD (n = 6). Differences between *COX7RP*-Tg and WT mice were analyzed using a two-tailed Student's t-test. *P < 0.05.
