# Supplementary material for: Mitochondrial Respiratory Supercomplex Assembly Factor COX7RP Contributes to Lifespan Extension in Mice
Source: Aging Cell. 2025 Nov 18;25(1):e70294. doi: 10.1111/acel.70294 (PMC12740103; doi:10.1111/acel.70294)
Supplement: Supplementary file 6 — Figure S6: acel70294‐sup‐0006‐FigureS6.pdf. [file ACEL-25-e70294-s008.pdf]

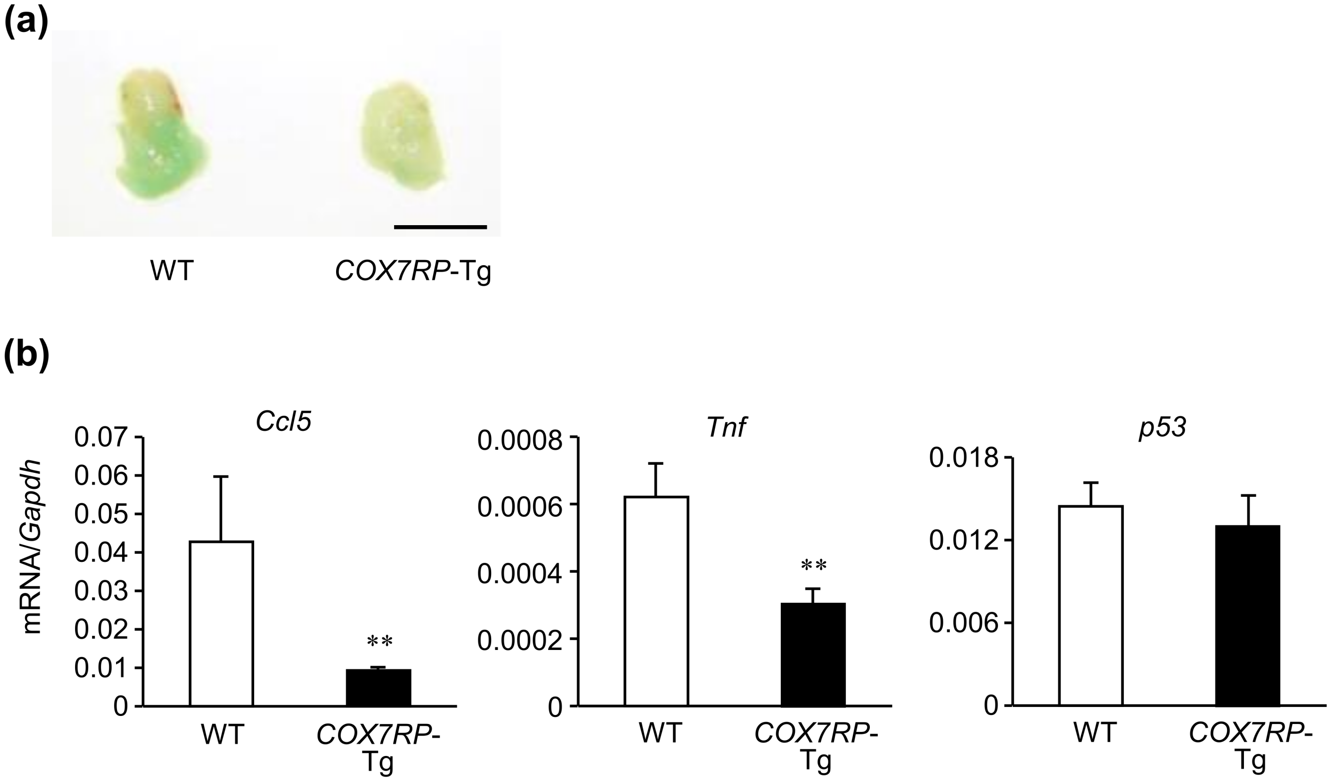

Figure S6 SASP-associated genes, *Ccl5* and *Tnf*, were downregulated in *COX7RP*-Tg WAT. (a) SA-βGal staining of WAT. Bar, 1 cm. (b) qRT-PCR analysis for *Ccl5, Tnf, and p53* expression in WAT of *COX7RP*-Tg and WT mice at 2 years old. Differences between *COX7RP*-Tg and WT mice were analyzed using a two-tailed Student's *t*-test. $^{**}P < 0.01$.
