# Supplementary material for: Mitochondrial Respiratory Supercomplex Assembly Factor COX7RP Contributes to Lifespan Extension in Mice
Source: Aging Cell. 2025 Nov 18;25(1):e70294. doi: 10.1111/acel.70294 (PMC12740103; doi:10.1111/acel.70294)
Supplement: Supplementary file 7 — Figure S7: acel70294‐sup‐0007‐FigureS7.pdf. [file ACEL-25-e70294-s002.pdf]

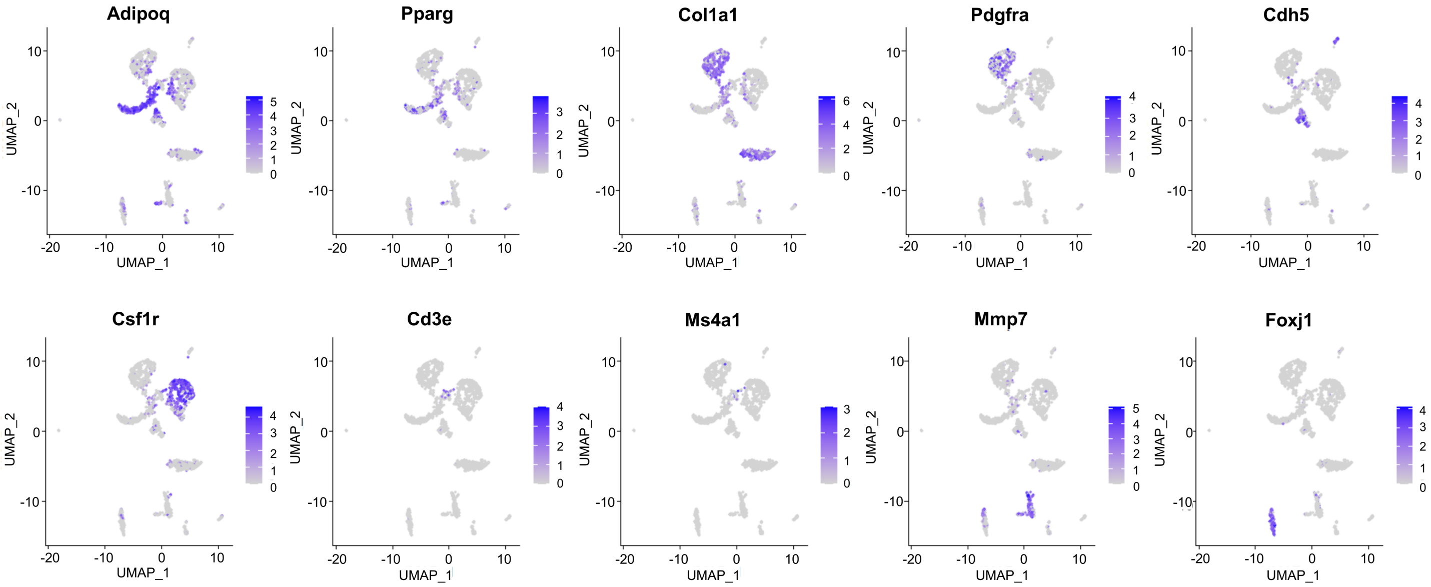

Figure S7 Uniform Manifold Approximation and Projection (UMAP) plots of snRNA-seq data in WAT based on cell type-specific marker expression. UMAP plots of snRNA-seq data for WAT obtained from young (8-week-old) and old (2-year-old) mice with either WT or *COX7RP*-Tg (Tg) phenotypes.
