# Supplementary material for: Mitochondrial Respiratory Supercomplex Assembly Factor COX7RP Contributes to Lifespan Extension in Mice
Source: Aging Cell. 2025 Nov 18;25(1):e70294. doi: 10.1111/acel.70294 (PMC12740103; doi:10.1111/acel.70294)
Supplement: Supplementary file 8 — Figure S8: acel70294‐sup‐0008‐FigureS8.pdf. [file ACEL-25-e70294-s009.pdf]

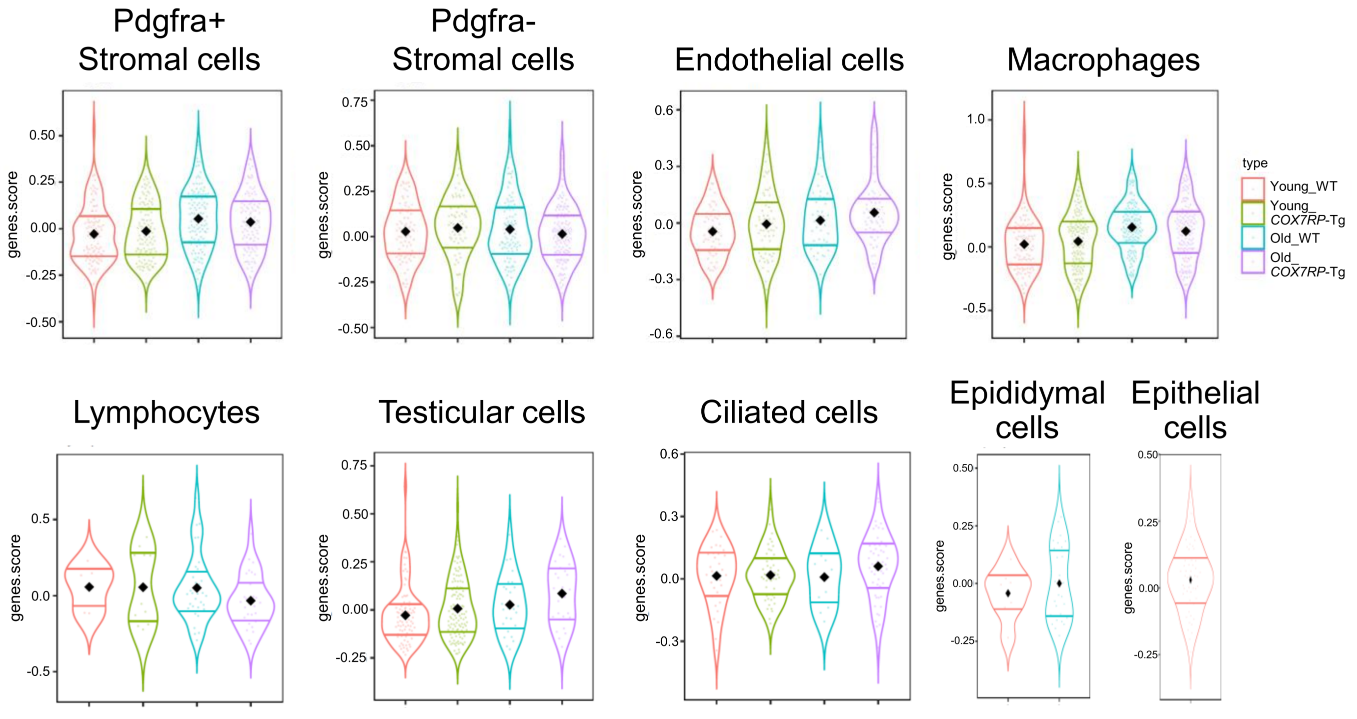

Figure S8 Violin plots for scores of SASP-associated genes in WAT cell clusters except adipocytes from distinct mice groups. Violin plots representing scores and expressions of selected SASP-associated genes in the indicated types of cells. The SASP-associated genes score was calculated from the sum of Z-scores for 18 SASP genes. A significance test was conducted using the one-way ANOVA followed by Tukey method, and *P* values were calculated for each group comparison. Differences between groups did not reach statistical significance.
