# Supplementary material for: Mitochondrial Respiratory Supercomplex Assembly Factor COX7RP Contributes to Lifespan Extension in Mice
Source: Aging Cell. 2025 Nov 18;25(1):e70294. doi: 10.1111/acel.70294 (PMC12740103; doi:10.1111/acel.70294)
Supplement: Supplementary file 9 — Figure S9: acel70294‐sup‐0009‐FigureS9.pdf. [file ACEL-25-e70294-s006.pdf]

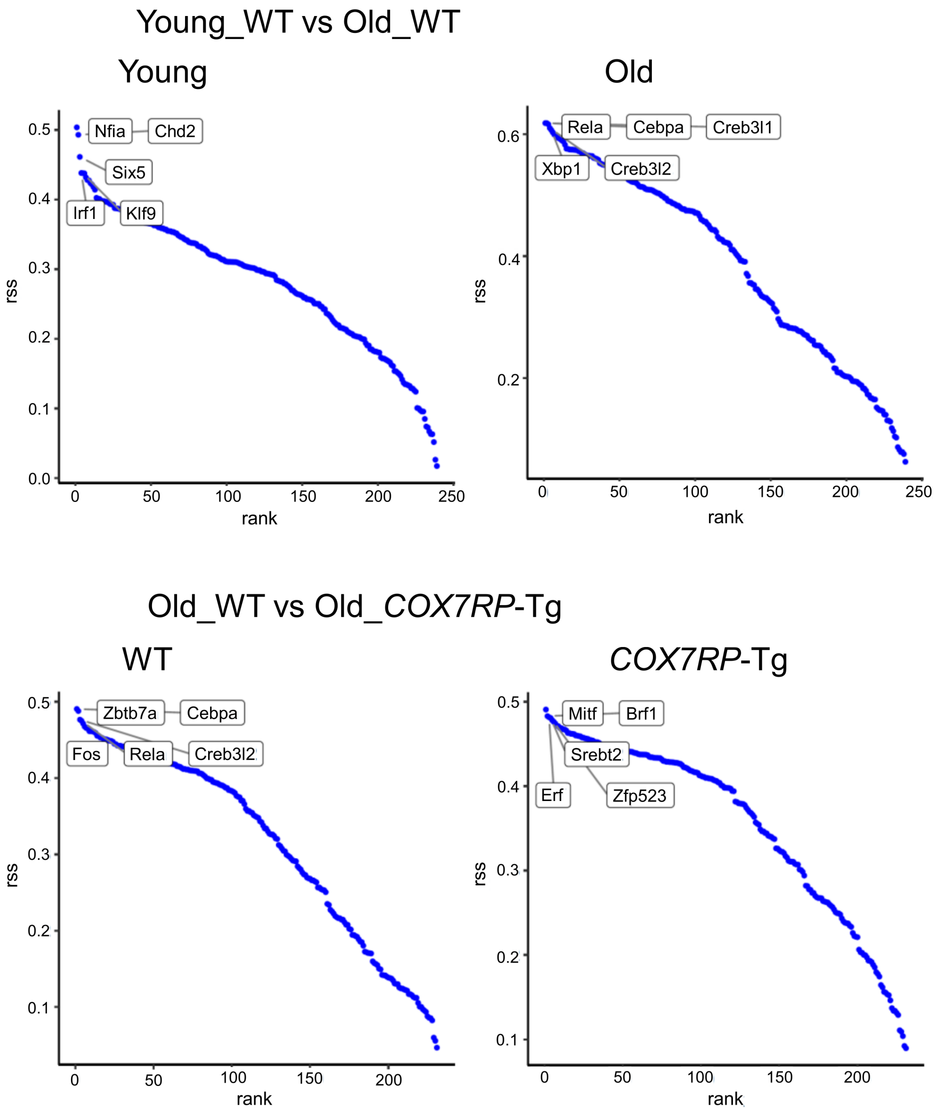

Figure S9 Rank of predicted upstream regulators for SASP-associated genes with significant motif enrichment in adipocytes from each mice group based on regulon specificity score (RSS) determined by SCENIC algorithm.
